# Review of Failure Mechanisms of Steel Wire Ropes Under Heavy-Load Conditions and the Anti-Friction Effects of Gel-like Grease

**DOI:** 10.3390/gels11110900

**Published:** 2025-11-10

**Authors:** Han Peng, Yihao Zhang, Linjian Shangguan, Minzhang Zhao, Bing Li, Leijing Yang, Yannan Liu

**Affiliations:** 1 School of Mechanical Engineering, North China University of Water Resources and Electric Power, Zhengzhou 450045, China; 18539131830@163.com (Y.Z.); david19881007@163.com (B.L.); 2 School of Water Conservancy, North China University of Water Resources and Electric Power, Zhengzhou 450045, China; 3 Zhengzhou Aote Technology Co., Zhengzhou 450045, China; zmz@autol.ne; 4 Henan Institute of Special Equipment Inspection Technology, Zhengzhou 450047, China; YLJ89932567@163.com (L.Y.); tjlyn6@163.com (Y.L.)

**Keywords:** heavy load conditions, inspection techniques, gel-like grease, steel wire ropes, failure modes

## Abstract

The failure behavior of steel wire ropes under heavy load conditions is a complex system involving the interaction of mechanical damage, lubrication status, and detection technology. Despite numerous studies, the existing literature seriously lacks a systematic framework to correlate the structural integrity and deformation behavior of gel-like grease and its central role in suppressing the critical failure modes (wear, fatigue, corrosion) of steel wire ropes. This review aims to fill this critical knowledge gap. By critically synthesizing existing studies, this paper explains for the first time how the microstructural evolution and rheological behavior of gel-like grease can ultimately determine the macroscopic failure process and life of steel wire ropes by influencing the interfacial tribological processes. We further demonstrate, based on the understanding of the above mechanism, how to optimize the detection strategy and design high-performance gel-like greases for specific working conditions. Ultimately, this work not only provides a unified perspective for understanding the system reliability of steel wire ropes but also lays a solid theoretical foundation for the future development of intelligent mechanism-based lubrication and predictive maintenance technologies.

## 1. Introduction

### 1.1. Steel Wire Ropes Overview

Steel wire ropes are flexible, spatially helical structural steel products. Steel wire ropes are flexible ropes twisted from multiple steel wires, usually consisting of a steel wire, a core, and a lubricant [1]. The basic structure consists of wires, strands, and core [2]. Steel wire is the basic unit of wire ropes and is usually made of high carbon steel with high strength and abrasion resistance [3]. Strands are made by twisting multiple wires together, and the twist direction of the strands can be either left or right [4]. The core is located in the center of the steel wire ropes to provide support and lubrication, and common core materials include fiber core (FC) and steel core (IWRC) [5].

Steel wire ropes can be divided into lifting wire ropes, elevator wire ropes, mining wire ropes, marine wire ropes, and so on, according to their applications [6]. According to the structure, it can be divided into single-twisted steel wire ropes, double-twisted steel wire ropes, and multi-twisted steel wire ropes. According to the flick direction, it can be divided into right interactive twist, left interactive twist, right isotropic twist, and left isotropic twist [7]. Steel wire ropes manufacturing process is also an important part of steel wire ropes, mainly including raw material selection, drawing, heat treatment, twisting, and lubrication [8]. The manufacturing process of steel wire ropes selects high-carbon steel as raw material to ensure the high strength and abrasion resistance of the steel wire, and then the billet is drawn into the required diameter of the steel wire, the steel wire is heat-treated to improve its mechanical properties, and then, the steel wire is twisted into a strand, and the strand is twisted into a rope [9]. Finally, the steel wire ropes are lubricated during or after the twisting process to minimize friction and wear [10].

In the 21st century, the acceleration of infrastructure construction, deepening of energy development, and the expansion of the transportation sector on a global scale, together promote the booming development of the steel wire rope industry. Against this background, the Chinese market has witnessed explosive growth, and a number of leading enterprises with international competitiveness, such as CMG and Baosteel Group, have emerged; at the same time, the global market has also been dominated by international giants, such as Teijin (Japan) and Brugg (Switzerland), presenting a pattern of diversified competition. In recent years, under the guidance of China’s “Made in China 2025” and other industrial policies, and in conjunction with the general trend of the global manufacturing industry to intelligent and green transformation, the industry has accelerated the upgrading of high-end, intelligent and green direction, the product structure from the ordinary steel wire ropes to the high-strength, corrosion-resistant, and customized demand for the global high-end supply chain.

At present, the demand for steel wire ropes in global industrial production and infrastructure continues to grow [11], and the market size maintains a steady expansion. This trend shows a synergistic development in the East and West markets: on the one hand, in Asia, especially China’s “new infrastructure” policy drive, high-speed rail, wind power, ultra-high voltage, and other fields of high-strength, fatigue-resistant steel wire ropes surge; on the other hand, Europe and the United States, through the “Rebuilding for a Better Future” program and the “Green Deal for Europe” and other initiatives to promote a new generation of infrastructure for high-performance steel wire ropes purchases demand. The second is the rise of the global ocean economy, from China’s deep-sea aquaculture to the North Sea oil and gas platforms, from the South China Sea undersea cables to the Gulf of Mexico offshore wind power, all kinds of marine engineering scenarios to promote high-performance corrosion-resistant steel wire ropes demand to enhance more than 30%; third, the global smart manufacturing upgrade, whether it is Germany’s Industry 4.0, Japan’s robotics strategy, or China’s Manufacturing Power Plan, have spawned a miniaturization of the steel wire ropes, and customized demand for high precision steel wire ropes products. In this context, the steel wire rope failure mechanism research and its lubrication technology have attracted the attention of a wide range of research workers around the world, including Europe, North America, and Asia; a number of research institutions are in this field to invest a lot of research resources. Steel wire ropes, as an important load-bearing component, are widely used in construction, transportation, lifting, mining, ocean-going, and other heavy-duty working conditions around the world by virtue of their strong load-bearing capacity, excellent bending flexibility, and smooth and quiet characteristics during movement [12,13]. The specificity of the above applications makes the safety of steel wire ropes particularly important. Therefore, the failure mechanism analysis and lubrication technology research on steel wire ropes has important engineering value and theoretical research significance.

### 1.2. Consequences of Failure of Steel Wire Ropes

Steel wire ropes are subjected to repeated pulling, bending, and torsion loads for a long period of time under heavy load conditions [14,15]. While in contact with pulleys and reels, steel wire ropes may wear due to friction, and this abrasion accelerates the formation and expansion of cracks, thus leading to failure [16]. Steel wire ropes may fracture individual wires due to overstretching, bending, or impact, and this fracture is also the most common form of failure. Fracture can be subdivided into ductile fracture and brittle fracture, where fatigue is the main cause of steel wire ropes failure, which usually originates at the surface of the steel wire strands and propagates perpendicularly to the longitudinal axis [17]. In addition, when the steel wire ropes are in a wet environment or contact chemical media working conditions, they are often prone to corrosion. This corrosion causes the strength of steel wire ropes to be compromised, which in turn, enhances their likelihood of fracture [18]. Therefore, the main forms of steel wire ropes failure are wear, fatigue and corrosion [19]. The failure of steel wire ropes will bring a series of consequences: the failure of steel wire ropes will accelerate its obsolescence, resulting in the need to replace it in advance, thus increasing the cost of the project; the failure of steel wire ropes may lead to safety accidents, especially in lifting and hoisting operations, which may lead to the fall of heavy loads, resulting in injuries, deaths, injuries, and equipment damage, and other serious consequences; once the failure occurs, the consequences are unimaginable [20,21]. In order to ensure that the steel wire ropes can be operated safely, regular maintenance and testing are required, and the methods mainly include lubrication, appearance inspection, and nondestructive testing [22,23]. Maintenance work mainly includes regular lubrication for steel wire ropes, so as to reduce the degree of friction and wear and tear; check whether there are broken wires, abrasion, corrosion, and other types of defects on its surface; the use of magnetic particle detection, ultrasonic testing, and other technical means; explore the defects within the steel wire ropes [24,25]. Therefore, it is necessary to review the current status of the research on the failure mechanism of steel wire ropes and discuss its next research, with a view of providing a theoretical basis for the research on steel wire ropes.

### 1.3. The Role of Steel Wire Ropes Lubrication Technology

**Figure 1:**
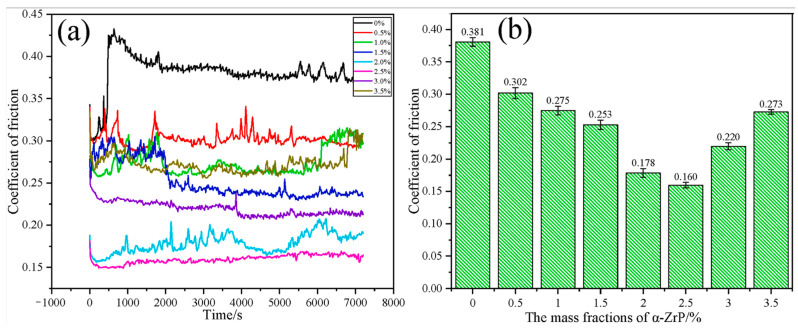
Variation curves of CoF of the wire ropes with time for the greases added different mass fractions of α-ZrP. (**a**) CoF; (**b**) average CoF [38].

The mechanical properties of steel wire ropes directly determine the smooth production [26]. However, steel wire ropes serve in harsh environments, complex working conditions, various forms of contact, there are lubrication failure and other injuries [27]. The specificity of the service environment of steel wire ropes brings many unstable factors to the mechanical properties of steel wire ropes, which seriously threatens the production efficiency and operational safety [28]. The role of steel wire ropes lubrication is reflected in the aspects of reducing wear and tear, preventing rust and corrosion, improving safety, prolonging service life, reducing maintenance costs and environmental protection [29]. The lubrication state of steel wire ropes has an important impact on the working characteristics and service life of steel wire ropes. In the past, academic researchers around the field of friction and lubrication, in order to ensure the safety and stability of steel wire ropes carried out many studies [30]. In the process of coal mine hoisting, elevator shaft hoisting, crane lifting operations, etc., the gel-like grease between the steel wire ropes is prone to deterioration, exacerbating the wear and tear of the wire ropes [31,32,33]. The central role of lubrication, particularly gel-like grease, in improving the tribological behavior of steel wire ropes has been widely confirmed by existing research. Its effectiveness is not only reflected in the base lubrication, such as McColl et al. [34], demonstrated that it can effectively reduce the coefficient of friction, but also significantly enhanced by the introduction of new additives. For example, the synergistic effect of nano and layered additives has become an important research direction: the studies of Brito et al. [35] and Jia et al. [36] have jointly shown that the friction reduction and anti-wear properties of materials such as carbon nanotubes and α-ZrP can even surpass those of traditional good materials, such as graphite and molybdenum disulfide, through the formation of more robust boundary lubrication films. These findings go beyond the importance of lubrication status alone as emphasized by Kishore et al. [37] and lead the research perspective to the “qualitative optimization” of lubricants rather than just “quantitative maintenance”, i.e., provide a clear path to enhance steel wire ropes life through material design. A clear path is provided to improve the wire rope life through material design. In the use of nano-additives to improve the performance of gel-like grease, there is a key “optimal concentration” phenomenon. The study by Zhang et al. [38] provides a typical example: they found that the α-ZrP additive is most effective at a mass fraction of 2.5%, too low to form an effective protective film, and too high to aggravate wear due to agglomeration instead. The results are shown in Figure 1, which demonstrates the influence law of the mass fraction of α-ZrP additive on the tribological properties of grease. This study provides an important basis for optimizing the formulation of grease and confirms that the optimization of the tribological properties can be achieved by precisely controlling the additive concentration.

The above research results show that steel wire rope lubrication technology by reducing the coefficient of friction, reducing wear and tear, preventing corrosion, effectively extend the service life of steel wire ropes, and at the same time, improve its operational efficiency and safety, reduce maintenance costs, and is of great significance to ensure the stable operation of steel wire ropes in complex working conditions.

Despite these advances, there is still a lack of a systematic review that specifically links the structural integrity and deformation behavior of gel-like greases to their performance in mitigating the major forms of wire ropes failure (wear, fatigue, corrosion). The novelty of this work lies in its comprehensive approach to fill this gap. It not only consolidates the current understanding of steel wire ropes failure mechanisms and lubrication technology, but more critically, it critically analyzes how the tribological properties of gel-like grease under different mechanical deformation conditions determine its effectiveness in extending wire rope life and ensuring operational safety.

In order to make up for this gap mentioned above, this paper firstly summarizes the failure mechanism of steel wire ropes and detection technology, discusses the reasons for the failure of steel wire ropes, the role of lubrication technology, and its development trend. The second part summarizes the three failure forms of steel wire ropes wear, fatigue, corrosion, and the influencing factors and the basic principles of wire rope failure detection methods and related research. The third part summarizes the effects of no lubrication, insufficient lubrication, and sufficient lubrication on the failure mode and serviceability of steel wire ropes, and reviews the tribological properties of the two types of wear, micromotion and sliding, of steel wire ropes under the lubrication conditions of gel-like grease. In the fourth part, the steel wire rope failure detection and analysis and gel-like grease lubrication research progress is summarized, and points to the future development of intelligent lubrication and predictive maintenance research direction. This paper aims not only to consolidate the existing body of knowledge, but also to provide a structured framework and forward-looking perspective that can drive the future development of the field.

**Figure 2:**
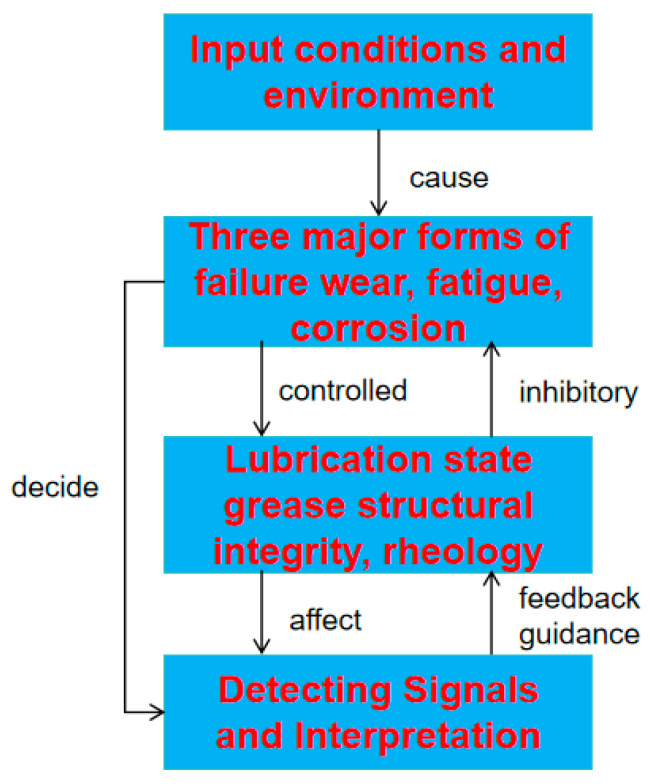
Associated framework diagram of steel wire ropes failure, lubrication, and detection under heavy load conditions.

Based on this, this review establishes a systematic analytical framework to elucidate the intrinsic linkages and dynamic interactions among failure mechanisms, lubrication states, and inspection technologies (as shown in Figure 2). In the following section, the components are discussed in depth around this framework.

## 2. Failure and Detection Analysis of Steel Wire Ropes

Steel wire ropes occupy a key position in construction, transportation, lifting, mining, ocean-going, and other industries, and their failure is directly related to personal and property safety. When steel wire ropes are lubricated with gel-like grease, its failure mode is closely related to the characteristics of the gel-like grease itself (high viscosity, semi-solid, strong adhesion), the working conditions of the wire ropes (load, environment, form of movement), and maintenance methods [39]. Steel wire ropes lubrication failure is one of the core causes of the overall failure of the wire rope, there is a direct and close causal relationship between the two—lubrication failure will accelerate the wear and tear, corrosion, fatigue, and other damage to the process of the steel wire ropes, and ultimately shorten its service life, and even lead to rupture and other serious failure of the accident [40]. These damages that steel wire ropes can suffer during use may lead to premature breakage of the rope assembly, and failure analysis of steel wire ropes can help to monitor the evolution of damage to the wire ropes in order to determine the conditions of use and to prolong the service life of the wire ropes [41]. By analyzing the failure of steel wire ropes in different application scenarios, this chapter can understand the mechanical property changes of steel wire ropes in the process of use, reveal the technical difficulties of steel wire ropes in the design, manufacture, use and maintenance, so as to formulate effective preventive measures, provide a scientific basis for the maintenance and testing of steel wire ropes, and help to formulate the maintenance plan and testing standards [42,43].

### 2.1. Failure Modes

1. Wear failure

**Figure 3:**
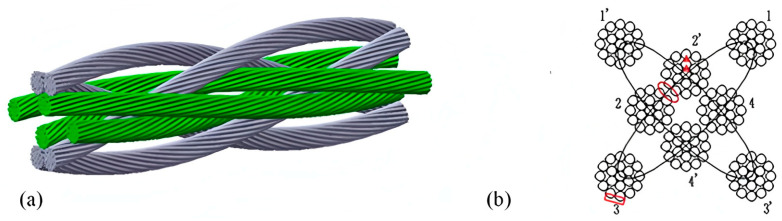
Details of the wire rope: (**a**) 3D model of the wire rope; (**b**) cross-section of the rope with the location of the different wear types [47].

Gel-like grease fills the small gaps between the wires, avoiding “dry friction” between wires and pulleys/reels and reducing the rate of surface wear. Although gel-like grease has strong adhesion, it will be gradually lost due to physical action under long-term working conditions, resulting in discontinuous lubrication film. Drying, hardening, and absence of gel-like grease can lead to steel wire ropes wear failure. Steel wire ropes wear failure is categorized into external, deformation, and internal wear [44]. External wear is the steel wire ropes are easy to contact with the pulley groove, reel wall, hook head, and other surfaces during the use of abrasion resulting in the diameter of the rope is thinned, and the fine steel wires on the peripheral surfaces are smoothed [45]. Deformation wear is localized wear on the surface of steel wire ropes due to vibration and collision [46]. Internal wear is the steel wire ropes in the process of use, due to the wire rope bending so that the internal fine steel wires interact with each other to produce slippage, strand to strand contact stress increases, and adjacent strands of steel wires between the local indentation deep concave [47]. The steel wire ropes model and different wear locations are shown in Figure 3.

Mechanical wear, rope core issues, single-sided wear, and compression wear can all cause the three types of failure mentioned above [48]. For example, prolonged mechanical wear, including wear on the surface of steel wire ropes and between the steel wires in each strand; core shrinkage and local shrinkage, core damage caused by impact force or exposure to flame spray; the steel wire rope runs on pulleys with rough rope grooves; steel wire ropes may fall outside the pulley or onto damaged rim areas during installation or when in a slack state [49,50,51].

2. Fatigue failure

Gel-like grease reduces friction stress on steel wires during repeated bending (such as when passing through pulleys), delaying the formation and propagation of fatigue cracks. Damage to the protective barrier provided by gel-like grease can cause uncontrolled fatigue damage to the wire rope, ultimately leading to fatigue failure. During use, steel wire ropes mainly undergo bending fatigue and fatigue caused by tension, torsion, and vibration, which is referred to as fatigue failure [52]. Fatigue failure occurs when microcracks form at the point of highest stress in the rope under cyclic loading, and then gradually develop, eventually leading to fatigue failure [53]. Steel wire ropes are subject to bending stress, friction wear, and excessive force, which cause stress concentration within the metal structure of the steel wire rope, leading to fatigue failure [54].

3. Corrosion failure

In acidic or alkaline environments, corrosive media react chemically with gel-based lubricants to form acidic or alkaline substances. These substances not only degrade the gel-based lubricant structure but also directly corrode the galvanized coating or metal substrate on the surface of the steel wire. Steel wire ropes exhibit three primary modes of corrosion failure: external corrosion, internal corrosion, and stress corrosion [55]. External corrosion is the most easily detectable form of failure. When the surface of steel wire ropes comes into contact with moisture, oxygen, and other media in the air, chemical reactions occur, resulting in rust formation [56]. This corrosion causes rust stains, rust scales, or rust spots to appear on the surface of the steel wire rope. As corrosion intensifies, the rust scales gradually peel off, exposing the steel wires underneath [57]. External corrosion reduces the surface finish of steel wire ropes and also reduces their cross-sectional area, thereby decreasing their strength. Internal corrosion is more hidden than external corrosion, but it is more harmful to steel wire ropes [58]. Moisture or corrosive substances penetrate through the gaps in the steel wire rope into the interior, causing corrosion of the steel wires inside the rope [59]. This corrosive degradation results in diminished tensile strength of the internal steel wires within the rope structure, and even the formation of corrosion pits in its interior; these corrosion pits will become a point of stress concentration, which is easy to induce fatigue cracking [60]. Internal corrosion may also cause the rope core to lose its supporting and lubricating functions, thereby reducing the structural stability of the steel wire rope [61]. Stress corrosion refers to the phenomenon of stress corrosion cracking of steel wire rope under the combined effect of corrosive media and tensile stress [62]. Corrosive media can penetrate into the interior of steel wire ropes, reacting chemically with the ropes. Under tensile stress, cracks form within the wires and gradually propagate [63]. This form of corrosion is more common in highly corrosive environments such as marine environments or chemical plants. Once stress corrosion cracking occurs, the strength of the steel wire rope will decrease sharply and may even lead to sudden rupture [64].

1. Wear Failure Mechanism

External wear occurs when steel wire ropes come into contact with the surfaces of objects such as pulley grooves, drum walls, and hook heads during use. The surface roughness and hardness of these contact points affect the degree of wear [65]. When the pulley groove is not smooth or the pulley does not rotate, the wire rope will experience one-sided wear during operation [66]. The material and surface treatment of objects that come into contact with steel wire ropes can affect the coefficient of friction. Higher coefficients of friction can lead to more serious wear problems [67]. Lubrication can reduce the coefficient of friction and wear [68]. Frequency of use also affects steel wire ropes, with frequent use accelerating external wear and tear [69]. When the crane repeatedly lifts and lowers heavy loads, the wear and tear of the wire rope in contact with the pulley and drum will increase [70]. In particular, steel wire ropes employed in mine hoisting systems operate under particularly demanding environmental conditions, and they are easily affected by vibration, poor lubrication, and mineral dust contamination, which can lead to increased friction and wear, and rapid deterioration of mechanical properties [71]. Deformation wear is caused by vibration and collision during the use of steel wire ropes, leading to localized wear [72]. For example, deformation and wear can occur when the steel wire rope on the surface of the drum is struck by other objects or when the lifting steel wire ropes of the crane become entangled with each other [73]. Equipment failures such as misalignment between the pulley and the drum center and uneven drum surfaces can cause localized compression and deformation of the steel wire rope, forming wings, hardening the surface material, and making it extremely prone to wire breakage [74]. In addition, improper installation or operation of steel wire ropes can also cause deformation, such as the steel wire rope jumping out of the pulley groove or drum guard, which can lead to local damage [75]. Internal wear occurs when the entire load is applied to one side of the steel wire rope as it passes through the drum or pulley, causing increased contact stress between strands and localized indentations and deep depressions in the wires between adjacent strands [76]. As a result of flexural deformation in the steel wire rope, the individual fine steel wires interact with each other, causing slippage and resulting in deep indentations in certain areas. When subjected to repeated cyclic stretching and bending, stress concentration occurs at these deep indentations, leading to fracture [77]. Wire ropes with line contact and surface contact have less internal wear than wire ropes with point contact because the wires in each layer of the strands of wire ropes with line contact and surface contact have equal twist distances and are in line contact with each other, thereby eliminating the secondary bending stress caused by point contact [78].

In practice, the wire rope will often appear around the groove stretch and torsion, in the long-term use of the state, which will make the rope groove interface contact fatigue, friction and wear and tear of the risk has increased [79]. The study of interfacial wear induced by relative motion in complex mechanical systems reveals that, although the objects of study are very different (from the macroscopic steel wire ropes-grooves to the fine engine fir-tree joints), their wear behavior is systematically governed by the mechanical state of the contact interfaces, with the “relative slip” being a common dominant factor. Chen et al. [80], in their torsional analysis of steel wire ropes wound around a groove, clarified that the interface wear is triggered by the slip component of the torsional motion. This finding echoes across the field with Lu et al. [81], who demonstrated through energy modeling that relative slip is equally critical in determining the wear mechanism and spatial distribution (e.g., the wear depth with respect to the position of the contact surfaces) in micromotor wear of aircraft engine joints. However, a profound contrast highlights the current modeling challenges: while Chen’s study focuses on macroscopic torsion under large-scale geometrical constraints (rope grooves), Lu’s model finely portrays the coupling effects of multiaxial loading (average load, stress ratio) and friction. This difference in scale and focus has led to the current lack of a unified wear prediction framework capable of accommodating both macroscopic geometric constraints and microscopic contact mechanics. Therefore, integrating mechanical models at different scales to accurately characterize wear due to slip in everything from steel wire ropes to precision components is a key direction for future research.

**Figure 4:**
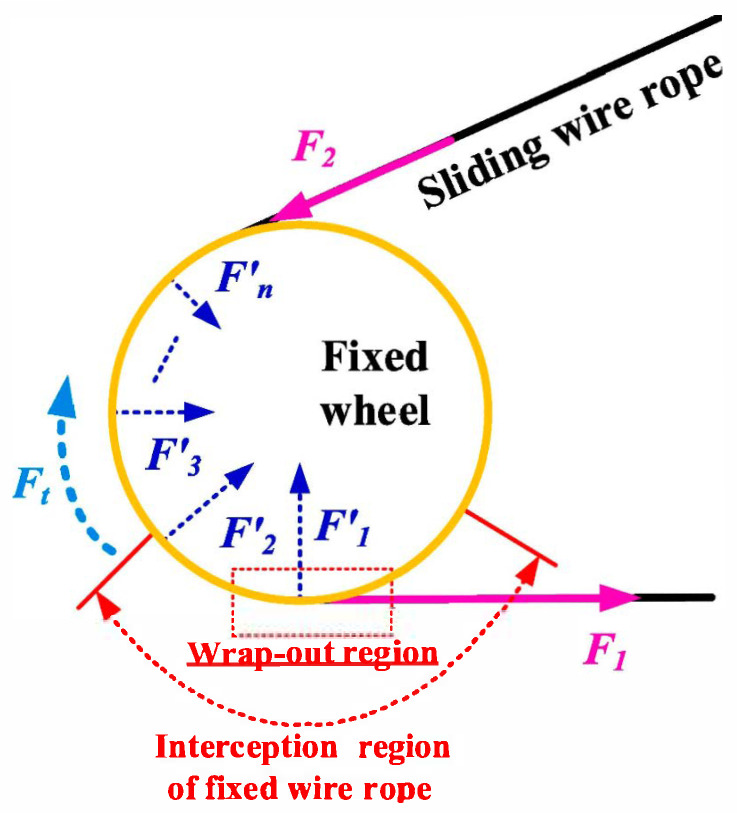
Force analysis of winding wire rope [82].

Winding traction conditions constitute one of the most demanding service environments for steel wire ropes, and its failure is the result of the synergistic effect of multiple stress modes such as pulling, bending, squeezing, and friction. In this context, the study by Zhang et al. [82] is of great value as it clearly reveals the complete evolutionary path of steel wire ropes from initial damage to final fracture. The study points out a key failure mechanism change: the fracture of unworn steel wire ropes begins in the internal stress concentration area (such as inter-strand extrusion and tension-bending coupling area), which belongs to the inherent fatigue weakness of the material; however, once the surface of the steel wire ropes are worn, cracks will be preferred to occur in the abrasion defects, and the failure mechanism is thus changed from “overall stress dominated” to “local damage dominated”, “change to” local damage dominance”. This shift is central to the predictive maintenance strategy: it means that the focus of monitoring and evaluation should be dynamically adjusted at different stages of the steel wire ropes’ life—early on, focusing on the overall stress distribution and internal defects, while in the middle and late stages, the focus needs to be on the evolution of the surface wear pattern. However, Zhang et al.’s study mainly separated the effects of tension, bending, and abrasion, and there is still a lack of quantitative description of the transient interaction of “tension-bending-friction” composite load, which is the most critical factor in twisted haulage, and its acceleration effect on the above mentioned failure paths. Revealing the damage acceleration mechanism under this multi-field coupling is the key to accurately predicting the remaining service life of steel wire ropes in wrapped haulage. The force analysis of the winding steel wire ropes under the simulated mine condition is shown in Figure 4.

**Figure 5:**
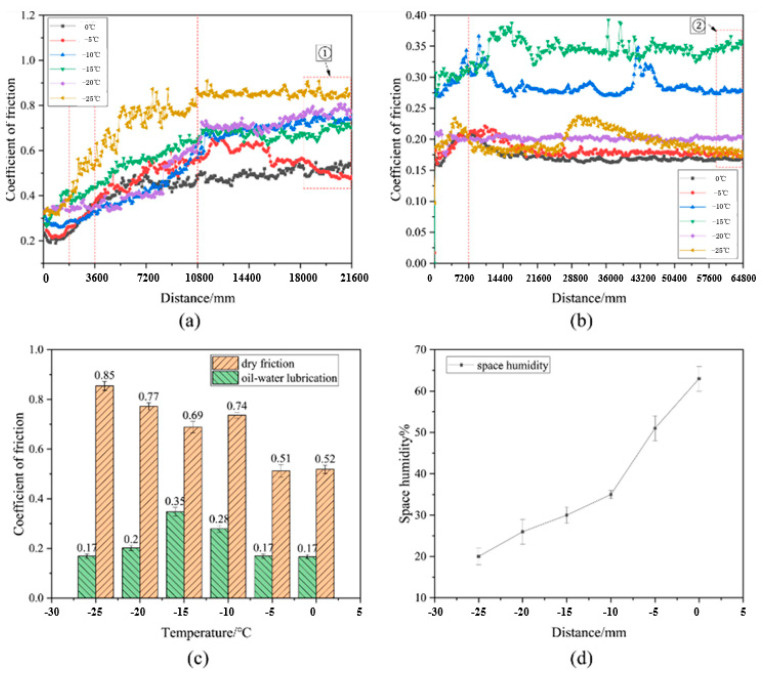
(**a**) Dry friction and (**b**) oil–water lubrication (v = 18 mm/s, F = 100 N); (**c**) friction coefficient of wire rope at different temperatures during stage ① and ②; (**d**) spatial moisture variations at different temperatures [84].

**Figure 6:**
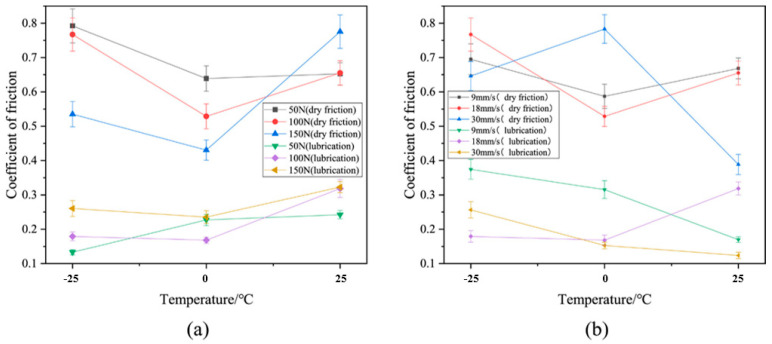
Variation curve of friction coefficient with environmental temperature under different loads (**a**) and different sliding speeds (**b**) [84].

Zhang et al. [83] investigated the friction coefficient and maximum friction temperature of steel wire rope in dry friction environment with the increase in tension force and rope speed, as well as the wear mechanism under different working conditions. The experimental data indicate a nonmonotonic variation in friction coefficient with increasing tension, initially decreasing before rising again, while exhibiting a linear decreasing trend with rope speed enhancement. However, Zhang et al. did not study the effect of temperature in the mine environment on the friction and wear characteristics of steel wire ropes. A study by Peng et al. [84] on low-temperature tribology revealed the decisive influence of temperature on the wear mechanism of steel wire ropes. As shown in Figure 5, the coefficient of friction shows a nonlinear variation with decreasing temperature under dry friction conditions, reaching a peak value of 0.85 at −25 °C. As shown in Figure 6, the tribological performance of steel wire ropes is significantly improved under oil–water lubrication conditions, and its wear area and depth are reduced by about three times and seven times, respectively, compared with the dry friction condition. It is worth noting that, although the lubrication state can effectively inhibit wear, but cannot completely eliminate the effect of temperature: the friction coefficient peaks at −15 °C (about 0.35), at which time the wear mechanism from oxidative wear dominant to adhesive wear dominant, reflecting the composite effect of low temperature and lubrication competition.

**Figure 7:**
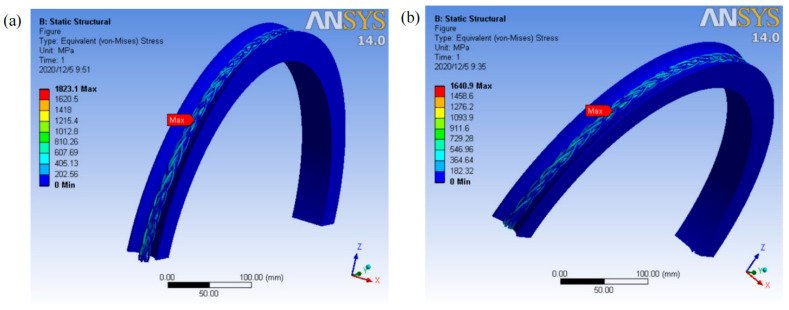
Total deformation of the same load with different friction coefficients. (**a**) 0.08, (**b**) 0.15 [85].

In terms of using numerical simulation to study the mechanical behavior of steel wire ropes, there is a significant difference in the rigor of model validation in existing studies, which directly affects the reliability of their conclusions and the guiding value of engineering practice. The study by Cui et al. [85] demonstrated the powerful capability of the finite element method in modeling complex working conditions (e.g., entangled hauling), and its parametric analysis revealed the critical influence of the inter-strand friction coefficient on the stress distribution of steel wire ropes, which provided important qualitative insights into understanding their internal mechanical mechanisms. The finite element analysis results are shown in Figure 7, when the friction coefficient is increased from 0.08 to 0.15, the stress concentration phenomenon is significantly improved, and the overall stress distribution is more uniform. However, a key limitation of the study is that the simulation model it developed lacks the necessary experimental data to validate it, which makes the quantitative accuracy of its predictions questionable. In contrast, the work of Ma et al. [86] systematically adopted a combination of experimental, theoretical, and simulation methods, and the experimentally measured stress–strain curves and ultimate strengths strongly verified the prediction accuracy of their finite element model, thus establishing the high reliability of their findings. The juxtaposition of these two studies profoundly reveals a general problem in methodology: despite the fact that finite element simulation can efficiently explore design parameters and operating conditions, an experimentally unvalidated model is still essentially a hypothesis to be confirmed. Therefore, future research must abandon the practice of relying solely on simulation and follow the paradigm of “simulation-experiment” cross-checking, which is the way to upgrade numerical analysis from trend guidance to accurate prediction.

2. Fatigue Failure Mechanism

Fatigue failure of steel wire ropes refers to the phenomenon in which microcracks form inside the steel wire rope under cyclic loading and gradually propagate, ultimately leading to broken wires or rupture [87]. Fatigue failure of steel wire ropes is usually related to factors such as load characteristics, material manufacturing processes, usage environments, and mechanical system design [88].

When steel wire ropes are wound around pulleys or drums, bending stresses are generated, the magnitude of which is related to the pulley diameter, steel wire rope structure, and position. Increasing the pulley diameter can effectively reduce bending stresses, thereby extending the service life of the steel wire rope [89]. When steel wire ropes come into contact with pulley grooves and drum walls, they generate compressive stress, leading to external wear and internal wear. Internal wear is particularly dangerous, as it causes stress concentration at the point of contact with the steel wire, accelerating fatigue failure. The steel wire is spiral-shaped, and tensile loads cause torsional shear stress on the steel wire, further exacerbating fatigue damage.

To investigate rotational speed effects, Onur et al. [90] performed comparative fatigue testing on both rotation-resistant and conventional wire ropes under bending conditions. They used a thermal imager to measure the heat generated by rotational speed and two pulleys of different diameters to measure the effect of pulley diameter on thermal deformation and bending fatigue life. Experimental data revealed a significant correlation between rotational velocity and the bending fatigue endurance of steel wire ropes. In addition, the fatigue life of steel wire ropes is closely related to the strength and toughness of the material. High-strength steel wire and optimized heat treatment processes can improve fatigue performance [91]. Coatings (such as zinc plating) can improve steel’s durability against both corrosive degradation and frictional wear wire, but excessive coating thickness can reduce fatigue performance. Studies have shown that when the coating weight is between 12 and 60 g/m^2^, the fatigue life of steel wire is significantly improved [92]. The stranding structure of steel wire ropes (such as point contact, line contact, and surface contact) has a significant impact on their fatigue life. Steel wire ropes with surface contact have the lowest contact compression stress and the longest service life. When steel wire ropes are used outdoors or in environments with harmful gases, corrosion pits easily form on their surfaces, becoming stress concentration points that accelerate the initiation of fatigue cracks. Extreme temperature variations significantly influence the mechanical performance of steel wire ropes, leading to notable degradation in fatigue resistance. In practical applications, optimal wire rope service life requires careful consideration of both pulley material properties and groove hardness characteristics. Pulleys equipped with nylon groove liners can effectively reduce wear and extend the service life of the wire rope [93]. Fatigue damage caused by reverse bending is twice that caused by forward bending, so the number of reverse bends in steel wire ropes should be minimized as much as possible.

To characterize interfacial damage mechanisms, Hu et al. [94] examined the mechanical response and wear evolution of steel wires in rope contact zones subjected to tensile-tensile fatigue loading. Through finite element simulations of wear progression across varying tensile stress ratios coupled with microscopic characterization, Hu et al. [94] identified maximum relative displacement and contact stress at the core strand-outer strand interface, correlating with severe localized wear evidenced by morphological and elemental analysis. The contact area between the strands had high iron, carbon, and chlorine content, as well as plastic deformation, delamination, and cracks, which led to stress concentration and reduced the fatigue life of the steel wire.

Through systematic bending fatigue testing of YS 9 − 8 × 19 woven steel wire ropes, Han et al. [95] established that the failure mechanism involves synergistic interaction between cyclic bending stresses and progressive surface degradation, with most wire breaks occurring in areas of severe wear. The most severe damage to adjacent strand wires is caused by bending friction fatigue. The wire–pulley interface primarily experiences fatigue-induced and abrasive wear mechanisms, whereas inter-strand contacts exhibit a more complex wear pattern combining fatigue, adhesion, and tribo-oxidation processes.

**Figure 8:**
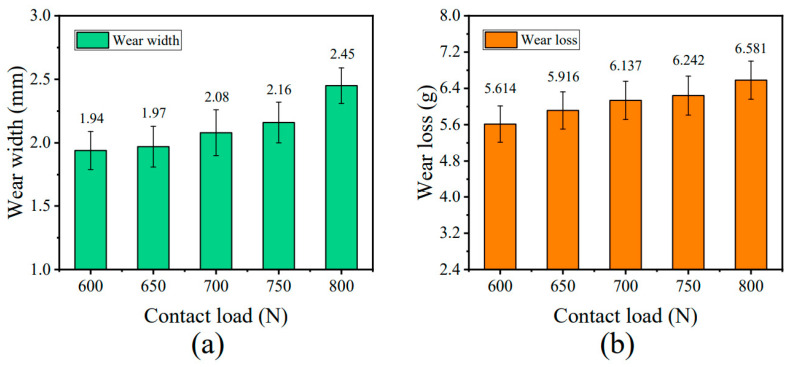
Wear characteristic parameters of wire rope under different contact loads: (**a**) maximum wear width; (**b**) wear loss [96].

The study by Chang et al. [96] revealed the coupled influence mechanism of contact load on the wear behavior and fatigue performance of steel wire ropes through systematic experiments. As shown in Figure 8, as the contact load increases from 600 N to 800 N, the wear width shows a nonlinear increase (1.94 mm → 2.45 mm) and a sharp increase in the 700 N–800 N interval, which reflects the synergistic effect of plastic deformation and wear of the material under high loads. It was also found that an increase in contact load resulted in a smoothing of the wear surface, indicating a shift in the wear mechanism from mixed wear to typical abrasive and adhesive wear. However, Chang et al. only studied the wear mechanism, ignored the wire rope volume, and did not study the specific location of wire rope wear, which is critical to the fatigue life of steel wire ropes.

Li et al. [97] studied the bending fatigue damage behavior of steel wire ropes in lifting systems. They constructed a multi-pulley test apparatus, calculated the friction coefficient using Euler’s formula, and observed the contour and morphology of the wear marks. Manually dismantled the wire rope to count the number of broken wires and found that the friction coefficient decreased from the fast end to the dead end, the abrasion mark sizes followed a normal distribution, and the broken wires were concentrated in the middle section of the wire rope. Therefore, fatigue damage first occurs in the middle section of the wire rope.

**Figure 9:**
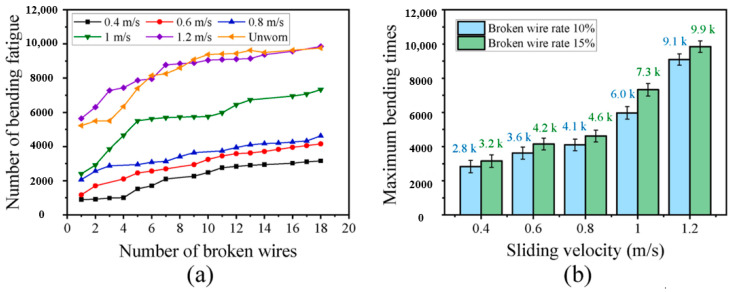
Bending fatigue properties of wire rope with different surface wear: (**a**) variation process of broken wires; (**b**) maximum bending fatigue times [98].

A study by Chang et al. [98] revealed a nonlinear correlation between sliding speed and fatigue life of transmission steel wire ropes. As shown in Figure 9, the flexural fatigue life decreases sharply from 9900 to 3200 h when the sliding speed is reduced, showing a significant velocity sensitivity. This phenomenon stems from the increased wear caused by the deterioration of the interface lubrication conditions at low speeds in combination with the increase in contact time, which leads to an accelerated degradation of fatigue properties. The study established the quantitative relationship of “sliding speed—surface wear—fatigue life”, which provides an important basis for steel wire ropes condition monitoring. However, the study has not yet resolved the differential effects of different wear morphologies on crack initiation, and it is suggested that subsequent work should establish a fatigue life prediction model that includes wear characteristics to enhance the accuracy of engineering assessment. These findings suggest that the frequency of monitoring the surface condition of steel wire ropes needs to be strengthened in low-speed and heavy-duty working conditions.

The center of gravity of the global elevator market has been significantly tilted to Asia, in which China is a big country in the production and use of elevators. According to the official data of Finland Tongli (Kone (Espoo, Finland)) website, China became the world’s largest market for new elevator installations in 2022, accounting for 62% of the global market for new elevator installations. However, the challenge of corrosion and high temperature and humidity faced by steel wire ropes in elevator shafts is a universal international problem. Ordinary elevators may be exposed to sunlight and rain prior to installation, which is common in several regions of the world with humid climates, and elevator wire ropes in poorly drained elevator shafts are often soaked during the rainy season. In addition, external elevators installed in older buildings around the globe often have widespread problems with moisture, shading, and heat dissipation in their shaft structures due to architectural and cost constraints, resulting in long periods of high temperatures and high humidity in the shaft. For example, such problems are particularly acute in regions with humid climates such as Southeast Asia and Western Europe, as well as in regions prone to condensation, such as the Middle East, where there is a large difference between daytime and nighttime temperatures.

Xue et al. [99] referred to existing methods and combined them with standards to propose an elevator wire rope fatigue test method. This approach involves simulating environmental circumstances like high temperature, constant temperature, and humidity, alternating damp heat, and acidic atmosphere to carry out tests. It can rapidly uncover how various conditions affect the fatigue performance of wire ropes, while also investigating their fatigue life and scrapping situations. Budrin et al. [100] put forward an approach to evaluate the service life of steel wire ropes on the basis of nondestructive testing data. This method is based on at least one control study of the condition of steel wire ropes to determine problem areas, their length, start and end coordinates, and the metal cross-sectional area at the control moment. The service life of steel wire ropes is computed using experimental data. Chen et al. [101] investigated the evolution of bending fatigue behavior and failure mechanism of single-stranded wire ropes with defects under cyclic bending loads. They constructed a finite element analysis model of single-stranded wire rope with internal defects on the basis of considering the effects of average stress and elasticity, compared the fatigue life of defective and nondefective wire ropes, and explored the effects of the radial position of the defects. The results of the study show that the minimum fatigue life of ropes with sectional defects occurs in the region of the defects, while this region is located in the contact area of ropes without defects; the defects change the distribution of fatigue life, and although they can locally improve the life, they reduce the load carrying capacity; the larger the distance between the defective layer and the neutral layer, the earlier the stress yielding will be, and the higher the risk of fatigue failure will occur.

Engineering cementitious composites (ECCs) feature ultra-high ductility and multiple crack resistance. High-strength stainless steel wire ropes (HSSSWR) boast both high tensile strength and excellent corrosion resistance. Wang et al. [102] proposed that engineered cementitious composites (ECCs), when reinforced with HSSSWR, have the potential to be used as connecting plates, permanent formwork, and reinforcement members in bridge deck systems. The results of bending tests showed that HSSSWR-ECC slabs with different HSSSWR reinforcement rates and ECC formulations exhibited good crack control effects and deformation capacity when subjected to bending moments.

Li et al. [103] carried out fatigue tests on 102 naturally corroded wires in service for 23 years and 84 wires with artificially prefabricated corrosion pits to investigate the fatigue performance of the corroded wires in depth, elucidated the significant roles of the degree of corrosion and the parameters of the corrosion pits on the fatigue life, and gave an effective fatigue life prediction formula. Li et al.’s findings are of great significance for assessing and predicting the fatigue performance of critical structural components such as bridge suspension cables. Milone et al. [104] reviewed the fatigue damage analysis of high-strength steel wire ropes (HSS) and pointed out that under typical exposure and loading conditions, such structural components, are prone to corrosion and fatigue damage in suspension or bridge cables, and conducted fatigue demand analysis and strength assessment.

3. Mechanism of Corrosion Failure

Corrosion failure is one of the most common forms of failure in steel wire ropes, primarily influenced by various external factors such as the environment, material and manufacturing processes, usage and maintenance, mechanical damage, and structural design.

In marine or salt spray environments, chloride ions can accelerate the corrosion of wire ropes; moreover, the higher their concentration, the faster the corrosion rate of the ropes [105]. A damp environment is one of the main causes of steel wire rope corrosion. Moisture makes contact with the surface of the steel wire, triggering an oxidation reaction that results in corrosion. Acidic and alkaline environments exacerbate chemical corrosion of steel wire ropes, especially when the protective layer on the surface of the steel wire rope is damaged. The corrosion resistance of steel wire ropes is closely related to the composition of their raw materials. For example, stainless steel wire ropes with a higher chromium content have better corrosion resistance. In manufacturing processes, surface treatment processes such as galvanizing can effectively improve the corrosion resistance of steel wire ropes, but long-term exposure to corrosive environments may cause the coating to gradually deteriorate. Defects present during the manufacturing process (such as inclusions, decarburization layers, etc.) can serve as initiation points for corrosion, ultimately leading to the corrosion failure of steel wire ropes through various inducing factors. In terms of mechanical damage and structure, steel wire ropes may suffer surface protection layer damage due to mechanical damage (such as twisting or flattening) during use, thereby accelerating corrosion. The winding structure of steel wire ropes also affects their corrosion resistance; for example, tightly wound steel wire ropes are more prone to accumulating corrosion products, leading to exacerbated localized corrosion. In addition, high-temperature environments accelerate corrosion reactions and reduce the corrosion resistance of steel wire ropes. When steel wire ropes come into contact with different metals or are exposed to electric arcs, electrochemical corrosion may also occur.

In terms of assessing the corrosion resistance of steel wire ropes, the study by Karasu et al. [106] provides a comprehensive evaluation paradigm with a multi-method linkage, which systematically compares the corrosion behaviors of steel wire ropes of different structures in simulated marine environments through a combination of material mass changes, electrochemical testing (Tafel extrapolation), and mechanical property experiments. The comprehensive nature of the methodology is recognizable in that it goes beyond the evaluation of a single index to provide a more reliable basis for screening the types of steel wire ropes suitable for use in ports and ships in three dimensions: material loss, electrochemical corrosion rate, and ultimate mechanical property degradation. However, the study also exposed a common limitation in such evaluation systems: their conclusions are heavily dependent on specific accelerated experimental environments (e.g., full-immersion experiments or salt spray tests). Although these accelerated experiments are efficient for side-by-side comparisons, it is often difficult to accurately simulate complex factors such as alternating wet and dry conditions, ultraviolet irradiation, and alternating salt sprays at different concentrations in the real ocean atmosphere, which leads to possible deviations in the predicted long-term service life from the actual situation.

**Figure 10:**
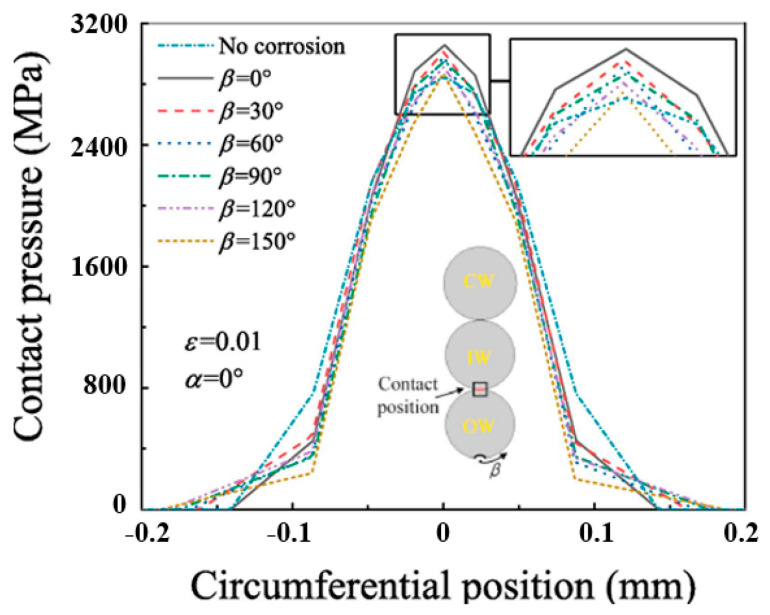
Distributions of the interwire contact pressure at different direction positions of the corrosion pit [107].

**Figure 11:**
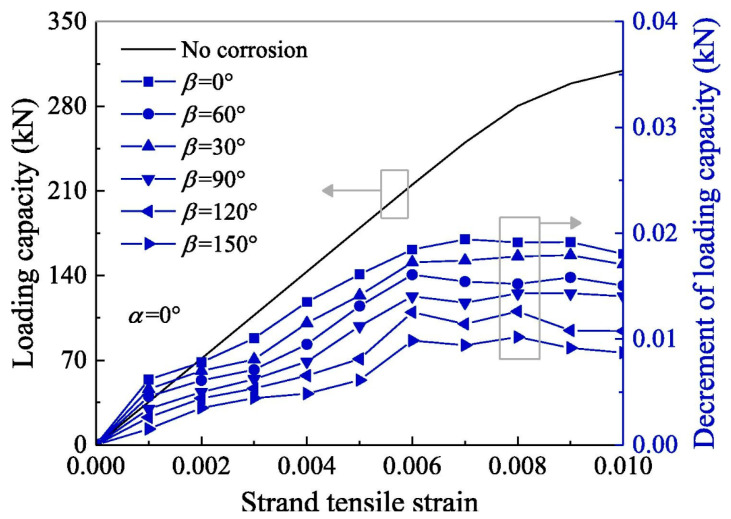
Loading capacity of the multi-layered wire rope strand at different position angles of the corrosion pit [107].

Chen et al. [107] explored the role of corrosion pits on the tensile mechanical properties of multi-layer steel wire ropes strands, derived the spatial parameter equations of the spools of corroded steel strands in the outer layer of the wire, and analyzed the effect of corrosion pits on the stress–strain, contact behavior, and load-carrying capacity of steel strands with the help of finite element simulation. The results are shown in Figure 10 and Figure 11. Figure 10 shows that corrosion pits lead to redistribution of contact pressure between rope strands, and when the long axis of the corrosion pit is perpendicular to the axial direction of the outer steel wires (α = 90°), the contact pressure reaches a peak, which greatly increases the risk of contact failure; Figure 11 further shows that the corrosion pits make the wire ropes’ load-carrying capacity generally decrease, and the loss of load-carrying capacity is the most serious when α = 90°. These findings confirm that the orientation of corrosion pits is a key factor affecting the degradation of steel wire ropes performance, and its harm is even more than the corrosion pit size itself. However, Chen et al. only simulated and analyzed the parametric equations, and the reliability of the simulation results needs to be further verified by corresponding tests in practical applications.

**Figure 12:**
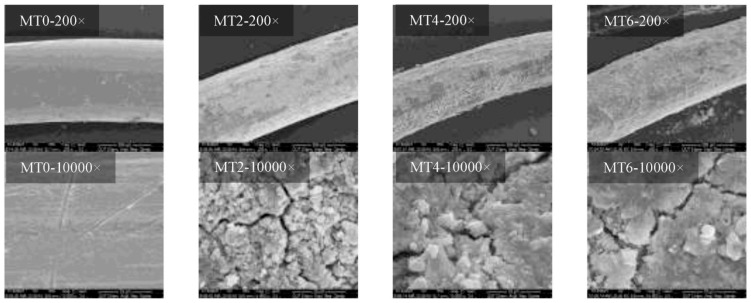
SEM images of wire rope specimens [109].

Youssef et al. [108] prepared specimens of 19 × 7 nonrotating steel wire ropes by accelerated corrosion with sulfuric acid and subjected them to static tensile testing. The study established a damage calculation model based on the static test data by fitting the fatigue law and then used the intersection of the reliability curve and the damage threshold to determine the predicted life of the steel wire ropes as a way to provide a critical time point for predictive maintenance. Yang et al. [109] conducted a study on the macromechanical behavior of round strand steel wire ropes in seawater corrosive environment. The study revealed the evolution of corrosion damage of steel wire ropes by scanning electron microscope system. As shown in Figure 12, with the increase in corrosion, the steel wire surface from the dense structure gradually developed into a rough morphology covered with loose oxides and microscopic cracks from the edge of the corrosion pit radial expansion, and ultimately triggered the material fracture mode from toughness to brittleness. The study established a complete correlation chain of “corrosion degree—microscopic morphology—macroscopic performance”, which confirms that the residual load carrying capacity of steel wire ropes can be effectively assessed by characterizing the surface cracks. However, current research has not yet established a quantitative relationship between microscopic crack parameters and macroscopic mechanical properties, and multi-scale models need to be developed to accurately characterize the evolutionary process from damage incipient to final fracture.

In the research field of steel wire ropes, explorations of their service performance degradation and pre-design modeling are often carried out independently, reflecting the separation of current research perspectives. On the one hand, Lin et al. [110] empirically demonstrated the critical role of grease on the corrosion resistance of steel wire ropes through systematic salt spray and mechanical tests. Its research reveals that effective surface oiling can nearly completely inhibit salt spray corrosion and maintain the mechanical properties of steel wire ropes. The discovery not only emphasizes that lubrication protection is indispensable but also points to a deeper challenge: the uncertainty of grease performance and its long-term effectiveness in harsh environments is still a bottleneck limiting the reliable application of steel wire ropes. On the other hand, at the front-end of design and analysis, Zhang et al. [111] worked on solving the efficiency challenges of geometric modeling, and they developed parametric software to achieve fast 3D modeling of steel wire ropes with shaped cross-sections. Although this work has significantly improved the design efficiency, the generated models have not yet been correlated with the service performance models such as corrosion and wear, which are the focus of Lin et al. Therefore, an obvious future direction is to combine Zhang et al.’s efficient modeling tool with Lin et al.’s service performance evaluation system to develop an integrated design-service digital twin framework that can simultaneously consider geometric, mechanical, and chemical factors, thus bridging the entire chain from accurate design to life prediction.

Finite element analysis by Hu et al. [112] showed that point contact steel wire ropes are more prone to failure due to stress concentration, while wire contact wire ropes have more uniform stress distribution and longer life. This reveals the core contradiction in steel wire ropes design: the trade-off between flexibility and fatigue life. What is more, the study found that the greatest stresses occur in the internal core, which poses a challenge to traditional inspection methods—how to monitor internal damage becomes a key challenge in achieving predictive maintenance.

In summary, the service life of steel wire ropes can be effectively extended and corrosion risks reduced by selecting materials with stronger corrosion resistance (such as high-chromium stainless steel) or improving surface treatment processes during the manufacturing process; regularly lubricating, cleaning, and inspecting steel wire ropes, and promptly repairing surface damage; and avoiding use in humid, salt fog, or chemical environments as much as possible.

### 2.2. Failure Detection Methods

As a key component in numerous large-scale devices, real-time monitoring of the operational status of steel wire ropes is vital for safeguarding lives and property [113]. Steel wire ropes gel-like grease lubrication state, directly affecting the failure of steel wire ropes detection method selection logic, detection accuracy and interpretation of the results—gel-like grease is not only the “protective barrier” of the steel wire ropes, but also may become a failure detection of the “Interference factors”: when the lubrication is good, the detection needs to focus on “early hidden failure”; lubrication failure, the detection needs to break through the “surface coverage interference”, and different lubrication state, the same under different lubrication conditions, the sensitivity and applicability of the same detection method may differ significantly [114]. For the different lubrication states of gel-like grease, it is necessary to target the selection of appropriate detection methods to ensure the accuracy of the detection—if the method does not match the lubrication state, it will lead to “missed/misdetected”, or fail to determine the nature of the failure [115]. At present, steel wire ropes failure detection methods mainly include appearance detection, nondestructive testing, mechanical performance testing, and intelligent detection technology.

The lubrication state has profoundly reconstructed the application logic and validity boundary of NDT technology, the core of which lies in changing the “signal conduction path” and “defect characterization interface”. In a well-lubricated state, a homogeneous grease film constitutes the first interface for inspection: it is transparent to magnetic fields (MFL) and stress waves (acoustic emission), but renders inspection based on the appearance of optics and machine vision completely ineffective. However, when the lubrication fails, the situation is reversed: the dried, hardened grease layer becomes a serious obstacle to signal propagation, which attenuates the magnetic flux and acoustic waves and produces pseudo-magnetic traces, greatly interfering with the accuracy and reliability of techniques such as MFL; however, at this point, the cleaned bare steel wire surface provides a clear observation window for visual inspection methods. This “one against the other” relationship reveals a key paradox: when grease needs to be monitored most for its protective effect (i.e., when it is on the verge of, or has already failed), its physical state interferes with the very core technology most often used to evaluate the object of its protection (steel wire ropes). This contradiction highlights the urgent need to develop intelligent detection algorithms that can adapt to or overcome lipid layer interference (e.g., signal compensation techniques for underlayers) or complementary detection strategies that incorporate multiple techniques. Appearance inspection is the most intuitive and basic inspection method. Through naked eye observation or with the help of a magnifying glass and other tools, check the surface of steel wire ropes for whether there are obvious defects such as broken wire, wear, deformation, corrosion, and so on. For example, check the surface of the steel wire ropes for signs of corrosion, whether there is a break in the steel wire exposed, and so on. Mnaka et al. [116] performed tensile, flexural, and torsional tests on 100 m long samples of the most worn steel wire ropes from a hoist to emphasize the importance of wire rope condition testing in a diagnostic system. The results showed statistically similar results for the different test methods, but the performance degradation was more pronounced in the bending test, suggesting that the current standard may need to be updated. This method is simple and easy to use, but it can only detect the more obvious damage on the surface and has limited effect on the detection of internal defects.

Nondestructive testing technology can detect the defects inside and on the surface of steel wire ropes without destroying the structure and performance of steel wire ropes, which is currently an important means of steel wire rope testing [117]. Nondestructive testing includes magnetic particle testing, ultrasonic testing, eddy current testing, and radiographic testing. Magnetic particle testing is a method that utilizes the magnetic properties of steel wire ropes to detect defects. When the steel wire ropes have cracks, broken filaments, and other defects, it will make the magnetic lines of force distorted at the defects, thus generating a leakage magnetic field. By applying the magnetic powder to the surface of steel wire ropes, the magnetic powder will be adsorbed at the defects under the action of leakage magnetic field, forming obvious magnetic traces, thus realizing the detection of defects. The state of gel-like grease lubrication directly affects magnetic field conduction and powder adhesion. Olszyna et al. [118] briefly described common methods of rope inspection to provide practical guidance for engineers. The case study in the paper demonstrates the advantages of magnetic methods in rope inspection for measuring changes in wire wear. The study addressed the problem of easy characterization and difficult quantification of external wire wear but did not detail the magnetic detection method or conduct tests to verify its usefulness.

Zhou et al. [21] proposed a surface broken wire failure analysis method combining finite element and mechanical tension, and found that the location of surface broken wire breakage in the use of lifting steel wire ropes is consistent with the maximum stress distribution, which verifies that the simulation matches the experiment. The accumulation and distribution of localized broken wires affect the residual strength, and this method analyzes the effects of different localized broken wire damages on the mechanical properties and provides a basis for the nondestructive testing of steel wire ropes.

The research of Zhang et al. [119] advanced the theoretical innovation in the field of nondestructive testing of steel wire ropes by combining the law of energy conservation with the J-A model to construct a force-magnetic coupling model under weak magnetic excitation, which provides a new analytical framework for realizing the quantitative detection of complex structural damage in steel wire ropes. Through the idea of “simulation first, experimental verification”, the study systematically extracted the characteristic values of the magnetic signals at the damage site, marking an important shift in the detection method from qualitative judgment to quantitative assessment. However, the practical application of the method still faces challenges: first, it is not clear whether the mapping relationship between the established magnetic signal characteristics and the damage type and depth is interfered by different lubrication states of the steel wire ropes (especially the thickness and homogeneity of the grease film); second, the stability of the model and the signal-to-noise ratio in the strong electromagnetic noise environment or under the conditions of high-speed moving steel wire ropes are still to be verified in the field. Therefore, the inclusion of workplace environmental variables in the applicability boundaries of the theory is a critical step in moving it from the laboratory to engineering applications.

**Figure 13:**
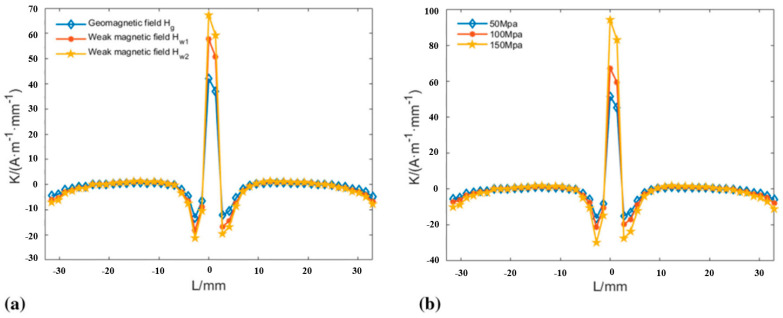
The distribution curve of the gradient value K: (**a**) different environmental magnetic fields; (**b**) different stresses.

Zhang et al. [120] confirmed a strong correlation between the characteristic parameters of the damage signals of steel wire ropes and the cross-section loss rate with the help of ANSYS 2022 R2 finite element analysis based on the metal magnetic memory effect. The study of Chen et al. [121] confirmed that weak magnetic excitation can effectively detect early damage of steel wire ropes. As shown in Figure 13, the detection method based on the principle of force-magnetic coupling is able to pinpoint the defect location by the peak value of the gradient parameter K, and the value of Kmax is positively correlated with the applied stress. This technique breaks through the dependence of traditional magnetic detection on strong magnetic fields and provides a new way to develop quantitative diagnosis of microdamage. However, the model simplification and insufficient samples of the study limit its engineering applicability, and it is necessary to improve the detection reliability by establishing a real defect model and improving the noise suppression algorithm.

In the field of nondestructive testing of steel wire ropes, the research is developing from the traditional theoretical model, device optimization to intelligent diagnosis and application of specific working conditions, and other levels of depth, in order to jointly improve the reliability, economy, and adaptability of the test. Ren et al. [122] are committed to solving the problem of high cost and complex structure of inspection equipment, and their designed magnetic focusing device can effectively improve the defect detection sensitivity through finite element simulation verification, which provides a valuable reference program for the development of low-cost, high-performance inspection system. Further, at the frontier of data processing and intelligent diagnosis, Han et al. [123] introduced a deep learning network based on residual modules, which confirmed the excellent performance of residual networks in complex internal and external filament breakage identification by comparing traditional BP neural networks, SVMs, and CNNs, and demonstrated the great potential of intelligent algorithms to realize high-precision and automated quantitative detection. Aiming at the shortcomings of the traditional magnetic dipole theory in quantifying internal defects, Zhao et al. [124] developed a leakage analysis model based on equivalent magnetic charge, which realizes the accurate resolution of different geometrical defects, and its simulation and experimental errors are controlled to be within 3% and 10%, respectively, which provides a key theoretical tool for the quantitative evaluation of defects. However, the model shows limitations when facing high-speed conditions, and as pointed out by Zhou et al. [125], the magnetization unsaturation induced by the velocity effect leads to signal distortion. To this end, Zhou’s team designed a detection device based on the principle of multiplicity, successfully realizing the effective identification of multiple broken wires at a speed of 3 m/s, which marks an important leap in magnetic leakage detection technology from static, low-speed scenarios to dynamic, high-speed applications.

Finally, in terms of closed-loop validation of the application of the technique, the work of Hu et al. [126] linked the above detection techniques to specific failure modes and verified the reliability of the damage assessment parameters by simulating the fatigue process of standing wave stacks and collecting signals. Their research not only provides methods for fatigue condition monitoring but also highlights a core need: any advanced detection technology (e.g., [120,121,122,126]) must be deeply integrated with the specific failure mechanism of steel wire ropes (e.g., fatigue breakage) for its assessment indexes to be of real guiding significance.

Critically, despite the above progress, the current research is still in a “silo” mode. The future breakthrough lies in the systematic integration of the accurate theoretical model of Zhao et al., the low-cost sensor of Ren et al., the high-speed solution of Zhou et al. and the intelligent diagnostic algorithms of Han et al. to build a next-generation inspection system that combines theoretical depth, economy, adaptability to working conditions, and intelligence.

The study by Krakowski et al. [127] extends the application of magnetic inspection technology to hybrid steel wire rope-belt load-bearing structures, and their design of a specialized measuring head provides a potential solution to address the wear assessment of such composite components. The importance of this work is that it reveals the adaptations and innovations that must be made to traditional steel wire rope inspection techniques when faced with new composite structures. However, the study mainly demonstrates the “possibility” of the application of the technology, the quantitative accuracy of its detection, the sensitivity of the mixed structure of the internal hidden damage of steel wire ropes (such as fatigue breakage), as well as the belt material itself on the degree of interference with the magnetic field, and other key issues, still require rigorous experimental data to support the systematic verification. Therefore, advancing the method from proof-of-principle to a reliable in-service inspection tool still requires in-depth research on signal-to-noise ratio enhancement and defect quantitative identification algorithms under complex working conditions.

**Figure 14:**
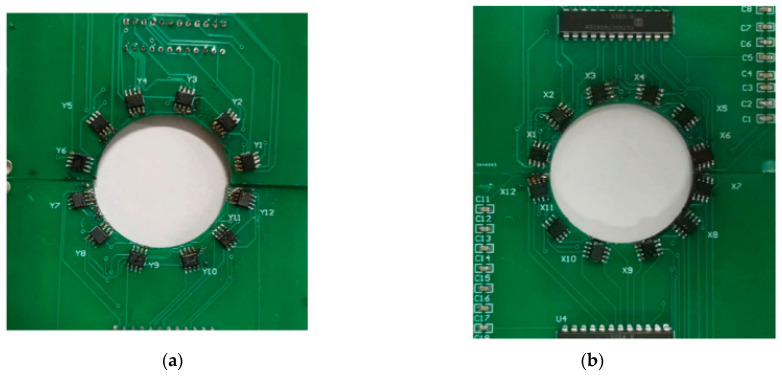

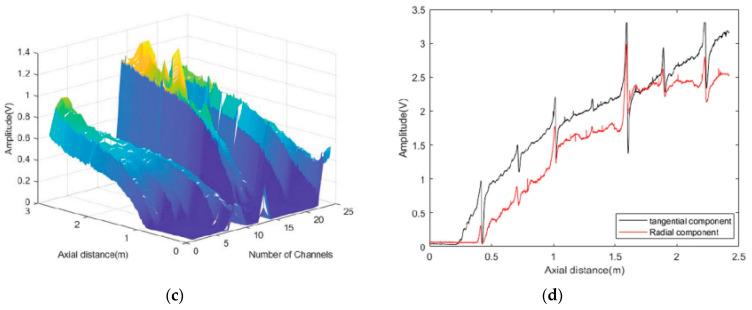
(**a**) Tangential sensor face, (**b**) radial sensor face, (**c**) raw magnetic, and (**d**) raw radial and tangential single-channel signals [124].

The wire ropes broken wire detection system developed by Chen et al. [128] achieves accurate identification and classification of damage by fusing 2D magnetic leakage signals with visible light images (Figure 14), combined with a filtering algorithm. The system innovatively integrates detection and processing functions to form a complete solution of “identification-localization-disposal”, which significantly improves detection sensitivity. However, the reliability of the system’s multi-sensor synergy under complex operating conditions still needs to be improved through field validation.

Yao et al. [129] developed an online intelligent nondestructive testing instrument suitable for mining steel wire ropes of various diameters, including an open-loop permanent magnet magnetization system, hardware peripherals, application software, and filtering algorithms. Experiments showed that the testing instrument can effectively identify broken wire defects in steel wire ropes of different diameters, with an average error of 0.36% in defect location detection. It has good application prospects. The test results are shown in Table 1.

Although the magnetized eddy current wall-climbing robot developed by Lu et al. [130] has made progress in automated inspection, its practical application still faces two major challenges: first, the robot’s lack of adaptability and reliability in harsh industrial environments, and second, the lack of quantitative comparative data with traditional methods to verify its performance advantages. The technology needs to undergo rigorous field validation and robustness enhancement before it can realize its true value for engineering applications.

Traditional one-dimensional (1D) and two-dimensional (2D) magnetic flux leakage (MFL) signal processing approaches applied to nondestructive evaluation (NDE) of steel wire ropes might result in missed detections and false positives, particularly under complex operating conditions. Liu et al. [131] put forward a novel MFL imaging and quantitative defect recognition method based on reconstructed sinusoidal function, wavelet transform, and mesh entropy matrix reconstruction. Additionally, they proposed and compared five distinct MFL imaging algorithms. In the case study and performance comparison, they developed and tested a quantitative assessment model for wire rope defect detection. In this model, the recognition results of six groups of wire rope detection signals not only validated the feasibility of the proposed quantitative method but also confirmed its effectiveness.

Huang et al. [132] addressed the issue of damage to steel wires used frequently in harsh environmental conditions by re-establishing mathematical models based on big data and developing the latest generation of wireless remote-controlled, real-time, dual-function steel wire nondestructive testing instruments using Intel chips. Although this method is effective for detecting surface and near-surface defects, it has relatively low sensitivity for detecting internal defects.

Aerial ropeways are widely used, and it is difficult to detect steel wire ropes for ropeways, and the cost of manual inspection is high and the safety coefficient is low. Although the deep learning algorithm proposed by Bao et al. [133] performs well in laboratory environments, its engineering application still faces a triple challenge: the model’s lack of generalization ability in complex natural environments, the susceptibility of visual detection to surface oil and light interference, and the lack of comparative validation with traditional methods. The technology must be rigorously tested in real industrial scenarios to prove its practical value [134].

Wang et al. [135] used dual-layer sensor differential signaling combined with an improved ResNet network to increase the accuracy of steel wire ropes damage detection to 94.05%. Although the method is effective in suppressing the lift-off effect, its engineering value is still limited by three aspects: the 3.15% accuracy improvement needs more scenarios to validate its engineering significance; the deep learning model relies heavily on the data quality; and the double-layer structure increases the complexity and cost of the equipment. This technology needs to find a better balance between data cost and detection accuracy.

Mechanical performance testing involves testing steel wire ropes through tensile tests, bending tests, and other methods to determine whether their strength, toughness, and other performance indicators meet requirements. For example, in tensile tests, parameters, such as the tensile strength and yield strength of steel wire ropes, are measured; in bending tests, the deformation and fracture of steel wire ropes during repeated bending processes are observed. Mechanical performance testing can directly reflect the load-bearing capacity and service life of steel wire ropes, but this method is destructive testing, which causes damage to the steel wire ropes and is generally only performed on a random basis when necessary.

With the continuous advancement of technology, some intelligent detection technologies have also begun to be applied in the field of steel wire rope failure detection. Intelligent detection technologies include fiber optic sensor detection, acoustic emission detection, and machine vision detection. Fiber optic sensors boast remarkable advantages, including anti-electromagnetic interference, strong corrosion resistance, and high sensitivity. Installing fiber optic sensors inside or on the surface of steel wires allows real-time monitoring of parameters such as stress, strain, and temperature. When defects occur in steel wire ropes, these parameters will change. By analyzing the signals transmitted by fiber optic sensors, abnormal conditions in steel wire ropes can be detected in a timely manner, enabling real-time online monitoring of steel wire ropes. Acoustic emission testing is a method of detecting defects based on acoustic emission signals generated by materials under stress. When there are defects inside a steel wire rope, micro-cracks or deformations may occur during loading, and these processes are accompanied by the generation of acoustic emission signals. By installing acoustic emission sensors around the steel wire rope to receive acoustic emission signals and analyzing and processing them, it is possible to determine whether the steel wire rope has defects and the development trend of the defects. Acoustic emission testing can monitor the dynamic changes of internal defects in steel wire ropes in real time during their use, providing important information for safety assessments. Machine vision testing uses computer vision technology to collect and analyze images of steel wire ropes. Images of steel wire ropes are captured using high-resolution cameras and then analyzed using image processing algorithms to identify defects such as broken wires, wear, and deformation on the surface of the steel wire ropes.

Based on the industrial needs of smart mines, steel wire ropes detection technology is developing synergistically along the paths of both algorithms and hardware, but its reliability from laboratory simulation to real-world underground applications still needs in-depth verification.

In terms of detection algorithm development, Yu et al. [136] focused on real-time requirements and designed a real-time detection system for broken wires. The value of the research lies in the confirmation of the performance advantages of specific algorithms in mining scenarios, which are capable of meeting real-time processing requirements. However, the evaluation environment of the system is relatively idealized, and the robustness of its algorithms in dealing with the actual working conditions such as complex electromagnetic interference, oil pollution coverage, and high-speed jitter downhole is still to be tested in the field for long-term operation. In terms of hardware platform and probe design, the study by Liu et al. [137] provides an important addition. By designing the simulation test platform and optimizing the C-shaped permanent magnet probe, they effectively solved the engineering pain point of traditional yoke-type equipment, which is bulky and fragile. The significance of this work is that it provides a more realistic experimental basis for algorithm validation. However, the authors also clearly point out that their research mainly relies on analog simulation and lacks durability test data in the harsh environment of a real mine.

Together, these two studies highlight a typical status quo in the current development of steel wire ropes inspection technology: algorithmic innovation and hardware optimization tend to advance independently. Therefore, the key direction for the future lies in the deep integration of advanced detection algorithms with robust hardware platforms and long-term, systematic comparative validation in real industrial scenarios as a way to cross the gap from laboratory performance to engineering reliability.

The combination of machine vision and deep learning provides an efficient solution for steel wire ropes inspection, and the studies by Zhou et al. [138] and Wang et al. [139] demonstrate the continuous progress of algorithmic iterations in improving inspection accuracy and functionality. However, there are fundamental limitations to the technology: its performance is heavily dependent on surface cleanliness and imaging conditions, and grease, oil, and light variations can significantly interfere with recognition. Current research is mostly validated in controlled environments, and its long-term robustness in real industrial sites needs to be verified urgently. In the future, it is necessary to break through the dependence on ideal images and build a more reliable hybrid detection system by improving environmental adaptability or fusing with detection technologies such as magnetic leakage. Table 2 synthesizes the research efforts of mentioned scholars, specifically detailing their methodological approaches to wire rope failure detection. The studies conducted by the aforementioned researchers are synthesized in Table 3, which presents a comparative analysis of different inspection methods for wire ropes.

In summary, the steel wire ropes inspection technology system is rich, from the traditional appearance of the inspection, magnetic leakage detection to the emerging machine vision, and intelligent identification, each has its own unique advantages and application boundaries. In order to systematically sort out the above methods and clarify their correlation with lubrication status and failure mode, this paper constructs the benchmarking system shown in Table 4. The table provides a systematic comparison of multiple dimensions such as sensitivity, limitations, cost, and applicability scenarios, and can be used as an important decision support tool for selecting the best detection solution in engineering practice.

### 2.3. Detection Technology Limitations and Field Application Challenges

Although various types of nondestructive testing techniques for the safety assessment of steel wire ropes provide a variety of means, but it must be recognized that there is no one technology is omnipotent, each method is subject to its physical principles, there are inherent limitations and application boundaries. Through the previous analysis, the main challenges can be summarized as follows: first of all, the principle of the technology itself exists in the perception of the blind spot, for example, magnetic leakage detection of defects parallel to the axis of the steel wire ropes is not sensitive to ultrasonic detection is heavily dependent on the coupling agent and the surface roughness requirements are extremely high. Secondly, field conditions introduce significant interferences, with the state of lubrication having a particularly strong influence: a homogeneous grease film obscures the machine vision, while a dry grease layer attenuates the magnetic and acoustic signals and is prone to produce pseudo-defect indications, leading to false positives or missed detections. Furthermore, economics and ease of use pose barriers to promotion; high-precision inspection systems (e.g., multi-channel magnetic leakage, acoustic emission) tend to be expensive, complicated to operate, and require specialized personnel to interpret the data, which limits their popularity among small and medium-sized enterprises. Therefore, in practice, the selection of detection methods must be weighed, and a strategy of one dominant technology supplemented by other methods of validation is often required to cope with complex and changing field environments.

## 3. Research Progress on the Role of Gel-like Grease on Steel Wire Ropes Wear Reduction

Steel wire ropes are an integral part of the global mining, construction, port lifting and transportation industries, and it has become an international engineering consensus to ensure their long life and safe operation, with lubrication and maintenance being a key component. Studies have shown that proper lubrication is critical because steel wire ropes are, in essence, a complex spatial mechanical system consisting of multiple strands of steel wire, not just a simple load-bearing cable [141]. This recognition has been clearly emphasized by international standards for lifting and transporting equipment such as UNE ISO 4308-1:2007 [142] and EN 12385-3:2020 [143]. Without effective lubrication, steel wire ropes are susceptible to corrosion, leading to internal steel loss, and this internal wear, which is difficult to detect from the outside, can significantly increase safety risks [140]. In fact, whether in the cold ports of Northern Europe, the high temperature of the Middle East’s oil fields, or the high humidity of Southeast Asia’s offshore platforms, the degradation of steel wire ropes caused by lubrication failure is a common challenge faced by the operation and maintenance of the equipment on a global scale.

### 3.1. The Effect of Gel-like Grease on the Performance of Steel Wire Ropes

Steel wire ropes are generally exposed to harsh outdoor conditions, where rust and corrosion frequently occur [144]. People generally regard steel wire rope as an industrial material, but in fact, it is composed of several moving components (steel wire, stranded rope, core, etc.). When steel wire ropes are subjected to tension, bending, and torsion, each wire and the core undergo friction and wear individually and in relation to each other [145]. Therefore, it is necessary to lubricate steel wire ropes to extend their service life and improve safety.

The lubrication condition is among the key factors that influence the performance of steel wire ropes. Excellent lubrication can notably lower the friction coefficient between the internal steel wires of a steel wire rope, as well as between the steel wire rope and external machinery. Reducing friction not only reduces wear, but also delays the formation and propagation of cracks, thereby extending the service life of steel wire ropes [146]. Conversely, insufficient lubrication can cause wear and tear on the surface of steel wire ropes, increasing the risk of wire breakage [147]. The lubrication condition exerts a notable influence on the bending fatigue life of steel wire ropes [148]. Studies indicate that under identical conditions, unlubricated steel wire ropes exhibit the highest number of broken wires, whereas fully lubricated ones have the lowest count of broken wires.

Peng et al. [149] clarified the composite failure forms dominated by adhesion, abrasive and fatigue wear under nonlubricated conditions, while Peng et al. [84] further revealed the differential effects of different lubrication states on the friction behavior. The recursive relationship between these two studies suggests that the understanding of wear mechanisms must move from a single operating condition analysis to a dynamic assessment framework based on lubrication status. However, these studies mainly focus on the static classification of the lubrication state and have not yet been able to reveal the dynamic correlation between the degradation of the gel-like grease performance and the evolution of the wear mechanism during continuous service, which limits its guiding value for the prediction of the wear of steel wire ropes in long-term service.

Xu et al. [150] confirmed that gel-like grease can significantly reduce the coefficient of friction between steel wires and optimize the stress distribution under the action of tensile-torsional coupling through micromanipulation wear test, which is of definite value for the enhancement of fatigue life of steel wire ropes. However, the study only verified the immediate effect of the grease and failed to explore its performance decay pattern due to mechanical shear and aging during long-term service. The exact correlation between this performance evolution and steel wire ropes life remains a key topic for future research.

Zhang et al. [82] established a steel wire rope vibration wear test platform and studied the vibration wear characteristics under fully lubricated and poorly lubricated (small amount of gel lubricant, dry friction) conditions. Through analysis of parameters, such as friction coefficient, temperature rise, wear depth, and volume, it was found that deterioration in lubrication conditions caused the wear mechanism to shift from fatigue wear to severe abrasive wear, adhesive wear, and fatigue wear. Under fully lubricated conditions, the vibration wear resistance of steel wire ropes was significantly improved. Zhang et al. only discussed the effects of lubrication conditions and vibration conditions on wire rope performance separately and lacked a systematic study of the coupling effects between these factors (such as the interaction between vibration and lubrication conditions).

For long-lasting lubrication, gel-like greases require a series of interrelated and sometimes constraining properties. The central challenge is to balance rheological properties with protection. On the one hand, the grease must have a wide range of high and low temperature properties (as indicated by the dropping point and low temperature brittleness point) and good adhesion to ensure that it does not lose or fail under operating conditions [151,152,153]; on the other hand, it also needs to have excellent rust and corrosion resistance [154]. However, these properties are often difficult to balance in practice. For example, greases thickened to improve high-temperature performance may have a concomitant reduction in low-temperature permeability and diffusion ability of rust inhibiting additives. Therefore, grease formulation design is essentially a performance trade-off process to find the optimal solution for a specific application scenario. At present, the anti-corrosion grease temperature and heat test method, the petrochemical industry standard “anti-corrosion grease salt spray test”, is recognized in the industry as a more appropriate test method to meet the performance requirements of steel wire ropes on the gel-like grease, often in practice due to the poor adhesion ability of the gel-like grease, in the use of the initial period of time caused by the loss of a short period of time or fall off, and play a role in the anti-corrosion. The advantages of gel-like grease lubrication are good lubrication effect, long duration, and not easy to lose. The adhesion and permeability of gel-like grease directly affect the lubrication effect, good adhesion can prevent the lubricant from being thrown off in high-speed movement or high-temperature environments, while good permeability ensures that the lubricant penetrates deep into the steel wire ropes and reduces the abrasion between the internal steel wires [155,156]. Harbor machine users and oil enterprise users require steel wire ropes gel-like grease has better adhesion to prevent the wire rope grease off to pollute the environment [157].

The friction-enhancing properties of steel wire rope gel-like grease are also extremely important. Mining friction wheel hoist to rely on the friction of the wheel, liner and rope to transfer power, to achieve the purpose of lifting, coal mine safety regulations put forward strong requirements [158]. Large-scale coal mine managers also pointed out that the use of poor-quality grease will be slippery steel wire ropes, and the removal of grease will lead to a decline in lifting performance, requiring a better friction-enhancing effect of the wire rope gel-like grease [159,160].

Golovin et al. [161] described the characteristics of Russian and foreign wire rope lubricants containing synthetic thickeners used by Russian wire rope manufacturers and provided comparative wear resistance test results for wire ropes lubricated with rope lubricants. The results show that, with the correct choice of lubricant, the service life of steel wire ropes can be increased significantly.

The environmental performance of gel-like lubricants for steel wire ropes is also a factor that cannot be overlooked during the application of steel wire rope lubricants [162]. According to surveys, manufacturers using steel wire ropes in elevators, cable cars, and similar applications have all raised environmental requirements. Customers for port machinery ropes and oilfield steel wire ropes all expect the ropes to be nonpolluting to the working environment. Elevator and cableway steel ropes require gel-like lubricants to be free of mineral resins to prevent environmental contamination and facilitate cleaning. During use, wire ropes cannot undergo heating and oiling as performed by manufacturers; only surface oiling and immersion are possible. Therefore, the grease must possess easy application at room temperature and excellent penetrating properties.

**Figure 15:**
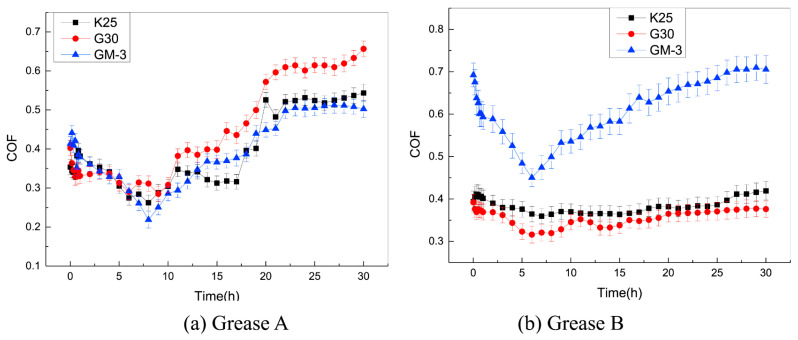
The coefficients of friction for a 30 h experiment (2 MPa) (**a**) the friction coefficients for the grease A (**b**) the friction coefficient for the grease B [163].

Feng et al. [163] revealed a positive correlation between loss modulus and friction coefficient of friction-enhancing greases, establishing a quantitative correlation between rheological properties and tribological performance. The results are shown in Figure 15, grease A decreases in viscosity due to temperature rise and the coefficient of friction decreases at the initial stage, but the coefficient rebounds after 10 h when the grease is extruded out of the interface to dry friction, while grease B remains stable. GM-3 liners are optimally adapted to grease A. The study shows that the sensitivity of the rheological properties of grease to temperature is a key factor in determining its frictional stability. This finding provides theoretical support for the improvement and research and development of new friction-enhancing gel-like grease, which is an important guiding value for the development of special greases for mine hoisting and harbor machinery.

The experimental study by Wang et al. [164] clarified the coupled effects of lubrication status and tension on the bending fatigue performance of steel wire ropes: adequate lubrication significantly optimizes the stress distribution and retards crack extension, while increasing tension destroys the integrity of the oil film and hinders grease penetration. This study reveals the interaction mechanism between the lubrication state and mechanical loading, which provides an important basis for the lubrication strategy under actual working conditions. However, its experimental design fails to deeply explore the quantitative relationship between tensioning force and lubrication deterioration, especially the critical threshold of oil film rupture and the consequent wear acceleration law has not been clarified. Therefore, modeling the quantitative correlation of tension force-lubrication state-fatigue life is a critical next step in translating this research finding into practical engineering guidance.

As mentioned above, fatigue is the primary failure mode of steel wire ropes under cyclic loads. Similarly, in humid and corrosive environments, steel wire ropes are prone to corrosion, and the lubrication status of steel wire ropes has a significant impact on both their fatigue performance and corrosion resistance [165]. Lubricants can fill microscopic cracks and defects on the surface of steel wires, reduce stress concentration, delay the initiation of fatigue cracks, and good lubrication reduces wear and damage to the surface of steel wires, thereby reducing the initiation points of fatigue cracks; lubricants can penetrate into the interior of fatigue cracks, forming an oil wedge effect, reducing the stress intensity factor at the crack tip, and inhibiting crack propagation. Lubricants possess a certain degree of viscoelasticity, enabling them to absorb vibrational energy and reduce the driving force for crack propagation; good lubrication can significantly extend the time it takes for fatigue cracks to start, which improves the fatigue life of steel wire ropes, improves the load distribution between steel wires, reduces local overloads, and delays fatigue failure [166]. Similarly, gel-like grease plays an important role in isolating corrosive media. Gel-like grease forms a protective film on the surface of steel wires, isolating them from corrosive media (such as moisture and salt spray). The lubricating film formed can block the ion channels required for electrochemical corrosion, slowing down the corrosion rate; some lubricants contain alkaline additives that can neutralize acidic substances in the environment and slow down corrosion. Corrosion inhibitors present in lubricants are capable of forming a chemical protective film on the surface of steel wires, thereby further delaying corrosion. Moreover, appropriate lubrication can notably prolong the service life of steel wire ropes in corrosive environments. Lubrication-based protection helps lower the maintenance and replacement expenses incurred due to corrosion [167]. The research of the aforementioned scholars is synthesized in Table 5, which summarizes their specific studies on the influence of lubrication conditions on wire rope performance.The lubrication condition not only affects the friction wear, fatigue and corrosion performance of steel wire ropes, but also has a comprehensive impact on their overall performance:

(1) Improved safety and reliability: Good lubrication reduces the risk of wire rope failure and improves its safety and reliability.
(2) Reduced operating costs: By reducing wear and tear and extending service life, maintenance, and replacement costs for steel wire ropes are reduced.
(3) Improved operating efficiency: Low friction coefficients and good lubrication conditions improve the operating efficiency of steel wire ropes and reduce energy loss.

**Table 5:** Describes the specific research conducted by researchers on the impact of lubrication conditions on steel wire rope performance.

Reference Number	Author(s), Year	Major Findings
[149]	Y, Peng. et al., 2016	Discovered the wear mechanism of steel wire ropes under nonlubricated conditions.
[84]	Y, Peng. et al., 2021	It was discovered that different lubrication conditions have different effects on steel wire ropes.
[150]	C, Xu. et al., 2025	It was found that under the same contact force, the friction force of unlubricated steel wire ropes was greater than that of water-lubricated ropes, while grease-lubricated ropes had the lowest friction force.
[82]	Q, Zhang. et al., 2025	It was found that the vibration wear performance of steel wire ropes was significantly improved under fully lubricated conditions.
[161]	V, Golovin. et al., 2022	It has been discovered that selecting the correct gel-like grease can significantly extend the service life of steel wire ropes.
[163]	C, Feng. et al., 2020	It was discovered that when the loss modulus of Zengmo gel-like grease is high, the coefficient of friction is also high.
[164]	S, Wang. et al., 2023	It was found that the better the lubrication condition of the steel wire rope, the lower the friction coefficient and the fewer broken wires.

Due to the widespread application of steel wire ropes in various fields, such as construction, mining, ports, marine engineering, elevators, and transportation, lubrication requirements vary significantly depending on the working environment. This section summarizes the lubrication characteristics and requirements of steel wire ropes in different application environments to guide the rational selection and application of lubrication technology. The results are shown in Table 6.

According to the above table, it can be seen that the selection of gel-like grease should be optimized according to the use of steel wire ropes and the working environment [168]. For example, mining steel wire ropes require lubricants with good friction-enhancing properties to prevent slippage, while wire ropes for elevators and ropeways require lubricants with good environmental properties [169]. The steel wire ropes working under high temperature conditions in the steelmaking metallurgical industry need to be lubricated with steel wire ropes gel-like grease with good high temperature performance, extreme pressure anti-wear performance, adhesion performance, and flame resistance [170].

In summary, the lubrication state has a decisive influence on the frictional wear, bending fatigue and corrosion behavior of steel wire ropes. However, the lubrication state itself is again dominated by the inherent properties of the gel-like grease. In order to realize the in-depth cognition from “phenomenon” to “mechanism” and to provide theoretical basis for accurate grease selection, it is necessary to further investigate the performance differences of different grease formulations and their evolution in long-term service.

First, the formulation system of the grease (including base oil, thickener, and additives) directly determines its core performance. For example, calcium sulfonate complex grease excels in marine corrosive environments due to its innate anti-rust and extreme pressure properties, while polyurea-based grease is the preferred choice for long-life lubrication of elevator traction steel wire ropes due to its excellent oxidative stability. In contrast, lithium complex grease shows good overall performance in high-temperature, heavy-duty mining conditions. This performance divide stems from the fundamental differences in strength, stability, and interaction with additives in the microstructures built by different thickeners.

Second, the performance of grease is not static, and its decay in long-term service is inherent in the eventual failure of the lubrication. Steel wire ropes run in the continuous mechanical shear will destroy the soap fiber structure of the grease, resulting in softening of the precipitation of oil; and thermal and oxygen aging will make the base oil deterioration, thickening agent hardening. These physical and chemical changes together lead to the loss of structural integrity of the grease and the degradation of the lubrication function, so that it is unable to maintain an effective protective film between the steel wires, which inevitably leads to the end of the increased wear and fatigue life decline.

Therefore, the understanding of grease must be extended from the static “performance parameters” to the dynamic “service life cycle”. Based on this knowledge, the following section will further delve into the behavior and mechanism of action of grease in specific forms of wear (micromotion, sliding).

### 3.2. Effect of Gel-like Grease on Micro-Motion Wear of Steel Wire Ropes

In the field of steel wire rope failure analysis and lubrication technology research, micro-motion wear of steel wire ropes under grease lubrication is an important area of study. Micro-motion wear refers to wear that occurs on contact surfaces during relative motion with small amplitudes. During operation, steel wire ropes are subjected to loads such as vibration, bending, and stretching, causing slight relative slippage between the wires and resulting in micro-motion wear [171]. Lubrication with grease is an effective protective measure that can significantly reduce micro-motion wear on steel wire ropes. Gel-like grease forms a lubricating film on the surface of steel wires, reducing direct contact between wires and lowering the coefficient of friction, thereby slowing wear. Under complex working conditions, steel wire ropes are prone to micro-motion wear, which significantly reduces their service life [172]. Micro-motion wear is usually accompanied by complex processes such as fatigue, corrosion, and oxidation [173]. Lipid lubrication is a common lubrication method that can effectively reduce the wear and tear of steel wire ropes, but its mechanism of action is not yet fully understood. Therefore, studying the micro-motion wear behavior of steel wire ropes under lipid lubrication has important theoretical and engineering significance. This section will focus on elucidating the mechanism, influencing factors, and current research status of micro-motion wear in steel wire ropes lubricated with gel-like grease.

In the wear-induced failure of steel wire ropes, the micro-motion wear occurring internally is of vital significance and also serves as a key factor in researching the wear failure of steel wire ropes. The study of micromotion wear of steel wire ropes has gradually developed from single-parameter analysis to a systematic exploration of multi-factor coupling. The studies of Xu et al. [174] and Huang et al. [175] laid the experimental foundation in this field: the former elucidated the laws of contact pressure and micromotion amplitude on the wear mechanism, while the latter realized the quantitative assessment of wear marks by introducing the confocal contour imaging technique, which pushed the research from qualitative observation to precise measurement. On this basis, Chen et al. [176] further considered the effect of crossing angle and developed a wear prediction model, marking a shift in research focus from phenomenal description to behavioral prediction. Wang et al. [177], on the other hand, developed a framework to correlate micromotion parameters with fatigue life, which for the first time, linked laboratory wear data to engineering life prediction. However, most of these studies have been conducted under idealized laboratory conditions, which are significantly different from the actual working conditions in the mine. Although Imran et al. [178] examined the effects of corrosive environments through finite element analysis, their model has not yet integrated the key mechanical parameters from the previous studies. Therefore, the core challenge of the current micromotional wear research is how to establish a unified model that can simultaneously couple mechanical parameters, environmental media, and service loads, so as to bridge the gap between laboratory research and the complex conditions of real mines.

Currently, most research on micro-motion wear is limited to the contact behavior between two steel wires. Research on multi-wire micro-motion wear under grease lubrication conditions and friction wear between ropes is still in its infancy.

The micro-motion wear of steel wire ropes is a complex physical and chemical process, which mainly involves various micro-motion wear mechanisms such as adhesive wear, abrasive wear, fatigue wear, and corrosion wear. There are also many factors that affect micro-motion wear in steel wire ropes, such as load, amplitude, frequency, and environment. Among these, the greater the load, the greater the contact stress between the wires, and the more severe the micro-motion wear; the greater the amplitude, the greater the relative sliding distance between the steel wires, and the more severe the micro-motion wear. The higher the frequency, the more micro-motion cycles occur per unit of time, and the more severe the micro-motion wear; humid, corrosive environments accelerate micro-motion wear of steel wire ropes [179].

**Figure 16:**
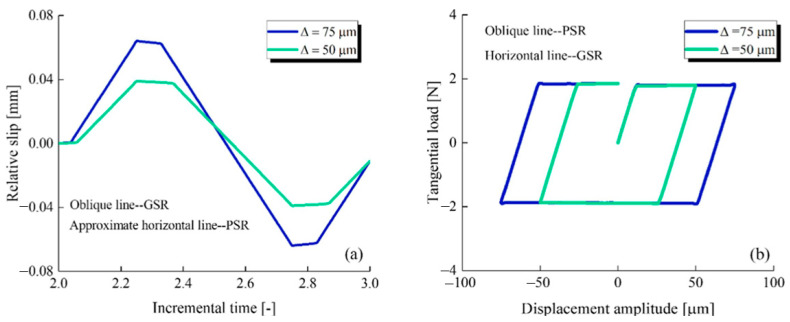
(**a**) Relative slippages and (**b**) hysteresis loops at different displacement amplitudes during one cycle [180].

Hu et al. [180] revealed the evolution law and protection strategy of micro-motion wear of steel wire ropes through a systematic study. As shown in Figure 16, the study clearly classifies the micromotion process into three typical phases: partial slip, mixed slip, and complete slip, and quantitatively confirms that the wear rate increases significantly with increasing slip amplitude. Hu et al. also proposed the use of grease, graphite, and other gel-like grease as well as the addition of hard coatings (e.g., DLC, WC-Co) can reduce its friction and wear; this discovery from the mechanism explains the reasons why the steel wire ropes are prone to early failure under vibration conditions. This study has constructed a complete theoretical framework of “micro-motion state—wear rate—protection method”, which provides an important basis for the selection of steel wire ropes protection strategy. Subsequently, efforts should be made to explore the synergistic protection mechanism of lubrication and coating in order to develop more efficient long-lasting protection technology.

The micro-wear of steel wire ropes under grease lubrication is influenced by multiple factors, primarily including the properties of gel-like grease, operating conditions, and rope structure [181]. The composition, viscosity, and extreme pressure anti-wear properties of gel-like greases all influence their lubricating effectiveness and resistance to fretting wear; load, amplitude, frequency, temperature, and other operating conditions affect the formation and failure of the lubricating film, thereby affecting micro-motion wear behavior; the structural parameters of steel wire ropes, such as wire diameter, number of strands, and twist pitch, affect the contact state and stress distribution between wires, thereby influencing the micro-motion wear of steel wire ropes [182]. Lubrication is an important means of reducing micro-motion wear on steel wire ropes. First of all, the gel-like grease will fill in the gap between the steel wire at the same time in the steel wire surface to form a layer of lubricant film. This film can separate the steel wires from each other, preventing direct contact between metal surfaces, thereby reducing adhesive wear and abrasive wear; in addition, the existence of lubricant film can also significantly reduce the coefficient of friction between the steel wire, reduce friction, and friction heat generation, in order to slow down the wear into. Secondly, grease lubrication can reduce friction heat and slow down material performance degradation. During the micro-motion process of steel wire ropes, friction between the wires generates a large amount of heat, causing local temperature increases. High temperatures accelerate oxidation and softening of the wire material, reducing its mechanical properties and making it more prone to wear. Grease has excellent thermal conductivity, effectively conducting friction heat away to lower the surface temperature of the wires and slow down material performance degradation [183]. Finally, gel-like grease can prevent corrosion of steel wire ropes and extend their service life.

Suspended mineral particles in mines accelerate wear on crane wire ropes, posing a serious threat to mine safety. Xu et al. [184] investigated the influence of different mineral particles on the fretting behavior between steel wires. Using a custom-built tester, they examined wire wear under various contact configurations. Results indicated that both particle type and concentration significantly affected rope fretting behavior: coal particles repaired wear at high concentrations, whereas ore particles consistently accelerated wear. Although mineral particles reduced wear, they accelerated wire failure. Therefore, caution is required when adding mineral particles to gel-like lubricants. However, the article does not present comparative studies on the lubrication effects of different particle size distributions, such as nanoscale versus micrometer scale. In humid, corrosive environments, the surface of steel wires is prone to chemical or electrochemical reactions, forming corrosion products. These products are removed during micro-motion, leading to material loss and accelerated wear. Gel-like lubricants isolate the wire rope from corrosive media, preventing corrosion and thereby extending the service life of the wire rope. Additionally, gel-like grease possesses certain viscoelastic properties that can absorb and dissipate vibration energy from steel wire ropes, thereby reducing micro-vibration amplitude and minimizing wear [185]. It can also fill gaps on the rope surface to prevent dust and impurities from entering, thus reducing the occurrence of abrasive wear.

Dyson et al. [186] demonstrated that micromotional wear is a key influence on the fatigue life of steel wire ropes through an innovative cross-cylindrical testing method, and established wear area increment as a core metric for evaluating the effectiveness of lubrication techniques. The value of this study is that it provides an experimental paradigm for quantitatively assessing lubrication performance, but the simplified contact model it employs differs significantly from the complex multiaxial stress state inside real steel wire ropes. Therefore, when applying laboratory-obtained lubrication key parameters to engineering practice, it is important to consider how well the scaling effect matches the actual working conditions. How to establish an accurate service life prediction model based on such basic test data is still an important topic to be solved.

**Figure 17:**
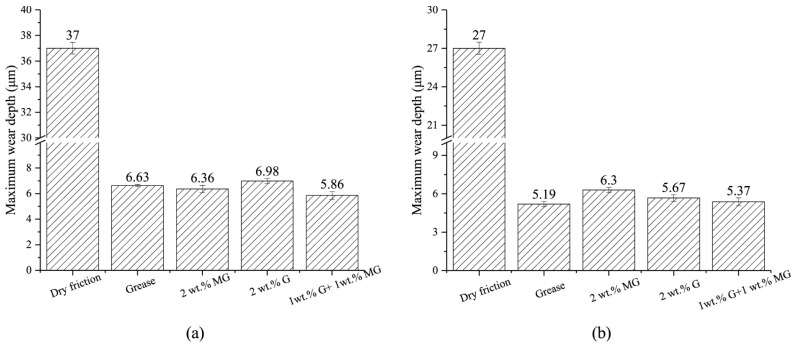
Evolution of maximum wear depth under different lubrication conditions for (**a**) the convex friction form and (**b**) for the concave friction form [187].

Sun et al. [187] systematically investigated the role of carbon-based additives in optimizing the performance of gel-like greases for steel wire ropes. Through a combination of four-ball friction test and homemade micromanipulation test rig analysis, it was found that the addition of either multi-layer graphene or micronized graphite can significantly enhance the anti-wear performance of the base gel-like grease IRIS-200BB. As shown in Figure 17, the composite formulation of 1 wt% MG and 1 wt% G has the most significant effect in reducing the micro-motion wear of steel wire ropes, and this optimal ratio provides a clear direction for the development of high-performance steel wire ropes special grease.

Wang et al. [188] found that the coefficient of friction was positively correlated with the temperature rise in multi-layer graphite grease lubrication, and this thermosensitive property is an important warning for lubricant selection under high temperature operating conditions. However, the study was limited to the observation of short-term friction behavior and failed to reveal the mechanism of the influence of this property on long-term wear and fatigue life. Subsequent studies need to focus on the structural evolution of graphite grease under sustained thermal loading and its quantitative relationship with steel wire ropes life.

Hoisting steel wire ropes are an important part of mine hoisting systems worldwide. Against the backdrop of deep and ultra-deep shaft mining becoming an international mining trend, from copper mines in Chile and potash mines in Canada to coal mines in Germany, wear and tear and damage to wrapped hoisting steel wire ropes are common technical challenges. Wound hoisting steel wire ropes used in China’s coal mines, especially those used in ultra-deep mines, are inevitably subject to abrasion and damage, which is highly consistent with the technological challenges faced by other major mining countries around the world. Practice shows that the use of good performance anti-wear gel-like grease is recognized by the international mining industry as one of the key technical measures to effectively improve the life of winding hoisting steel wire ropes, and the relevant lubrication specifications have been incorporated into ISO 6743-9:2003 [189] and other international standards guidelines.

**Figure 18:**
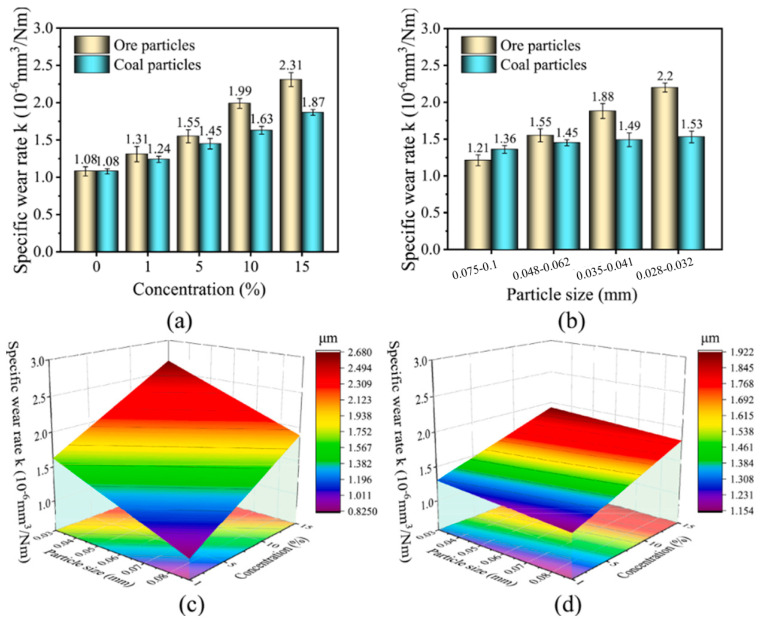
(**a**) Different ore particle concentrations, (**b**) different ore particle sizes, (**c**) specific wear rate evolution with ore particle concentration and size, (**d**) specific wear rate evolution with coal particle concentration and size [190].

**Figure 19:**
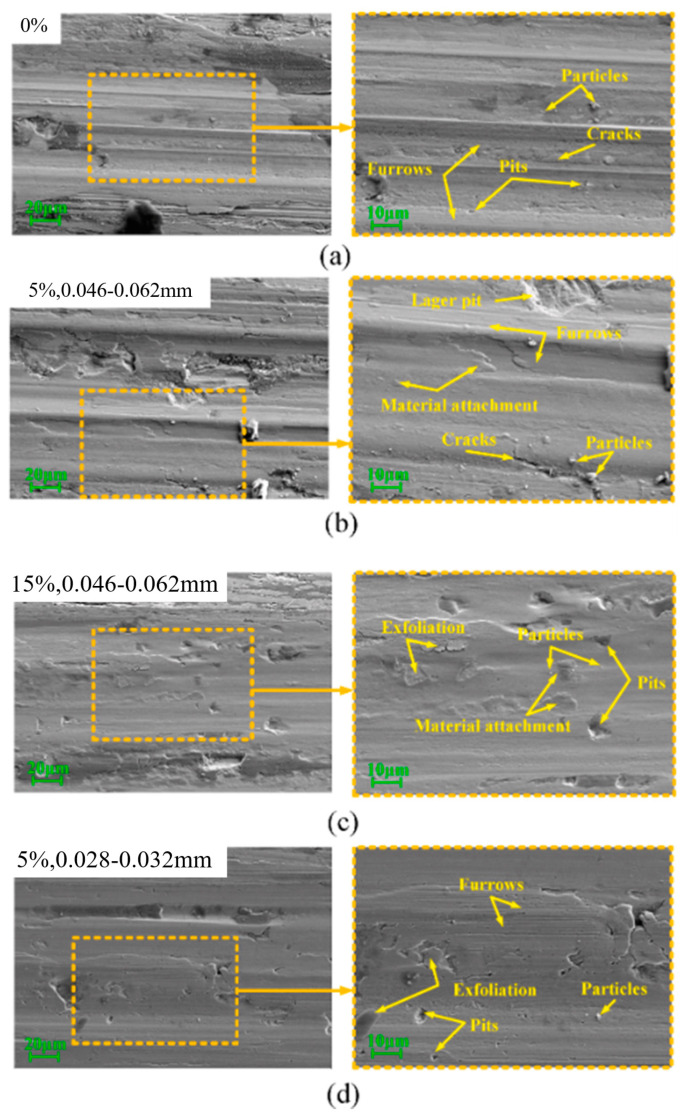
Morphology of worn surface after adding coal particles (**a**) Base grease (**b**) Grease with 5% ore particles (**c**) Grease with 15% ore particles (**d**) Grease with 5% small-diameter ore particles [190].

The study by Huang et al. [190] systematically revealed the significant effect of mineral particles on the micromotor behavior of steel wires through a homemade experimental setup. As shown in Figure 18, it was found that the addition of mineral particles to the grease led to an increase in the coefficient of friction and cumulative dissipated energy, where the coefficient of friction reached a peak value of 0.234 when mineral particles with a concentration of 15% and a particle size of 0.048–0.062 mm were added, and at the same time, the depth of wear (30.5 μm), the amount of wear (6706 × 10^3^ μm^3^), and specific wear rate (2.31 × 10^−6^ mm^3^/Nm) all reached their maximum values. Figure 19 shows that coal particles form a distinctive adhesion layer on the wire surface, accompanied by craters and spalling. Its low hardness and strong adhesion characteristics lead to a wear mechanism dominated by delamination and material transfer, which reduces cutting grooves but triggers localized stress concentrations and material stripping. This phenomenon reveals the decisive influence of the physical properties of the particles on the wear mechanism.

In summary, micro-motion wear of steel wire ropes under grease lubrication conditions is a complex multi-factor coupled process, the mechanism of which mainly involves the combined effects of lubricant film dynamic behavior, friction chemical reactions, and debris evolution: although the lubricating film formed by gel-like grease on the surface of steel wire can isolate the contact surface and reduce the coefficient of friction, it can become thinner or even break down under micro-motion shear and squeezing forces, leading to increased friction. Simultaneously, the reaction film formed on the wire surface through friction-induced chemical reactions with gel-like grease additives significantly influences wear characteristics. Moreover, the wear particles produced in the course of the wear process (which varies in size, shape, and hardness) has a dual effect: hard wear debris may cause abrasive wear, while soft wear debris may participate in the formation of a new lubricating layer. The interaction of these factors jointly determines the final form of fretting wear.

### 3.3. Effect of Gel-like Grease on Sliding Wear of Steel Wire Ropes

The wear and tear of steel wire ropes under sliding friction conditions has always been a focus of attention in the engineering field. Lip lubrication, as a simple and effective lubrication method, plays an important role in protecting steel wire ropes from sliding wear [191]. This section will elaborate on the mechanism of sliding wear in steel wire ropes lubricated with gel-like grease, its influencing factors, the mechanism of action of gel-like grease, and strategies for reducing friction and wear.

Under gel-like grease lubrication conditions, the sliding wear of steel wire ropes is a complex dynamic process involving the combined effects of multiple wear mechanisms. Compared with dry friction, under grease lubrication conditions, there is a grease film at the friction interface, and its wear mechanism exhibits some special characteristics, such as shear failure of the grease film, micro-asperity penetration of the grease film, and chemical action of the grease [192]. Shear failure of the grease film refers to the process in which the grease film is subjected to shear stress during sliding friction. Once the shear stress surpasses the strength limit of the grease film, the film will rupture, leading to direct contact between the steel wire rope and its mating parts, which in turn, gives rise to adhesive wear and abrasive wear [193]. Micro-protrusions can pierce through the grease film. Even in the presence of a grease film, micro-protrusions on the surfaces of the steel wire rope and its mating component may still penetrate the film, leading to local contact and consequently causing adhesive wear and abrasive wear [194]. The chemical action of gel-like grease affects it by enabling additives within the grease to potentially react chemically with the surface of steel wire ropes. This reaction forms a protective film, enhances surface hardness, and improves wear resistance [195]. However, certain additives may also react with the surface of steel wire ropes, accelerating wear and tear.

The sliding wear of steel wire ropes lubricated with gel-like grease is influenced by multiple factors, including the properties of the gel-like grease, lubrication method, lubrication cycle, and operating conditions. The viscosity, consistency, extreme pressure anti-wear properties, and other characteristics of grease directly affect its lubrication effectiveness and friction reduction and anti-wear properties [196]. Lubrication methods include manual application, drip lubrication, centralized lubrication, etc. Different lubrication methods affect the supply volume and uniformity of distribution of the lubricant, thereby affecting the lubrication effect. An excessively long lubrication cycle can lead to insufficient grease supply and reduced lubrication effectiveness; an excessively short lubrication cycle can result in grease waste and increased costs. Operating conditions such as load, speed, temperature, and contamination levels can affect the performance and lubricating effect of grease, thereby influencing the wear behavior of steel wire ropes [197].

Gel-like grease plays multiple roles in the sliding wear of steel wire ropes, with its primary mechanisms including friction reduction, wear resistance, cooling, sealing, and damping [198]. Gel-like grease forms an oil film at the friction interface, separating the steel wire rope from the mating components to reduce the coefficient of friction and minimize wear. Additives within the gel-like grease create a protective film on friction surfaces, enhancing surface hardness and improving wear resistance [199]. Lubricating grease can dissipate the heat generated by friction, prevent the steel wire rope from overheating, and reduce wear [200]. Grease prevents external contaminants from entering the friction interface and maintains lubrication. Grease absorbs vibration energy, reduces the amplitude of steel wire rope vibration, and reduces wear.

To effectively reduce sliding wear in steel wire ropes under grease lubrication, appropriate strategies are typically selected based on the application process of gel-like grease [201]. First, select the suitable type and grade of gel-like grease according to operating conditions. For instance, choose high-temperature grease for high-temperature applications and extreme-pressure grease for heavy-load conditions. Secondly, adopt reasonable lubrication methods and lubrication cycles to ensure that the friction interface is always in a well lubricated state [202]. Improving the structure of steel wire ropes can also effectively reduce wear. Optimizing structural parameters, such as the twisting method, number of strands, and wire diameter of steel wire ropes, can enhance their wear resistance [203]. Finally, surface treatment of steel wire ropes, such as coating and carburizing, is used to improve surface hardness and wear resistance. Additionally, we can develop new gel-like greases with enhanced anti-friction and anti-wear properties and extended service life, thereby effectively reducing sliding wear in steel wire ropes [39]. Research shows that grease lubrication can significantly reduce sliding wear on steel wire ropes. Most likes of steel wire ropes are coated with grease, and the steel wire rope fiber core is treated with hemp core grease. However, a few likes of steel wire ropes are not coated with grease due to usage conditions. Many steel wire rope manufacturers and users have noticed decades ago that lubrication conditions have a crucial impact on the service life of steel wire ropes [204].

**Figure 20:**
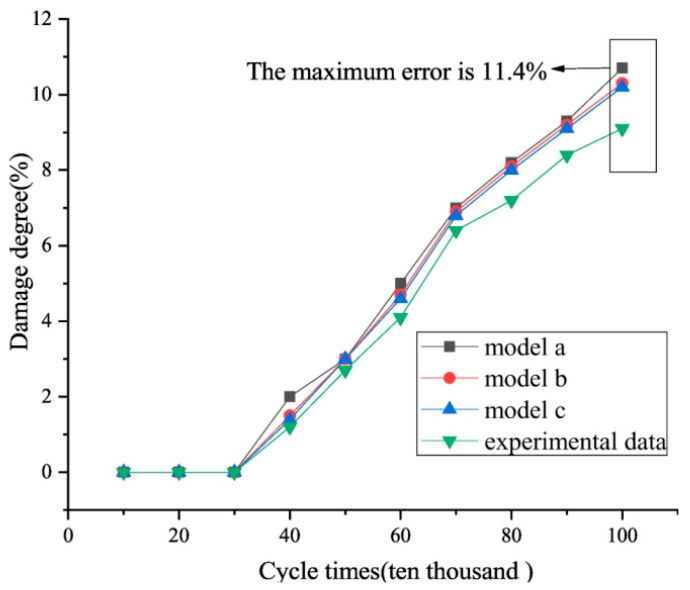
Damage degree contrast diagram [205].

Simplified computational model developed by Xue et al. [205] significantly improved the efficiency of fatigue damage assessment of steel wire ropes while maintaining the prediction accuracy. As shown in Figure 20, the model calculation results are in good agreement with the experimental data and successfully predict the wire breakage of steel wire ropes after 400,000 load cycles. However, the reliability of the model is significantly affected by the variation in friction coefficients due to lubrication conditions, and its generalizability to different engineering scenarios still needs to be systematically verified.

**Figure 21:**
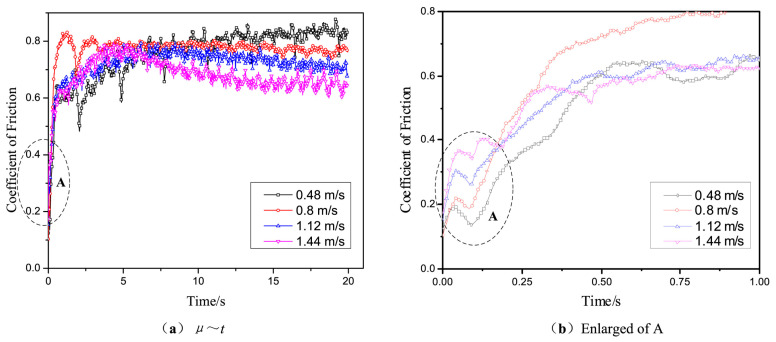
Variations in the COF of the wire ropes under different sliding velocities with impact for dry friction. (Impact velocity, 1.0 m/s; impact load, 600 N) (**a**) Coefficient of friction vs. time (**b**) Enlarged view of (**a**) [206].

**Figure 22:**
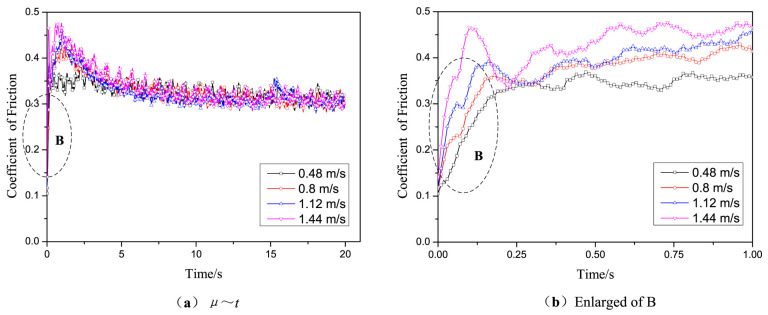
Variations in the COF of the wire ropes under different sliding velocities with impact for lubrication condition. (Impact velocity, 1.0 m/s; impact load, 600 N) (**a**) Coefficient of friction vs. time (**b**) Enlarged view of (**a**) [206].

Peng et al. [206] systematically revealed the key role of grease in the friction regulation of winch wire ropes by comparing the dry friction and lubrication states. As shown in Figure 21 and Figure 22, the steel wire ropes showed high friction coefficient (about 0.73) and significant temperature rise (103 °C) during dry friction, and the coefficient of friction with the increase in speed showed the typical characteristics of thermal softening; and after the use of IRIS-200BB grease, the coefficient of friction was stabilized at 0.35, which is a significant reduction of about 52%, and it is not affected by the change of load and speed. This study quantitatively confirmed the dual efficacy of grease in reducing friction and suppressing temperature rise and established a reliable basic database of dry friction and lubrication status. Although long-term performance studies were not involved, this work provides an important theoretical basis for the development of high-performance steel wire ropes special greases with interfacial stability and lays a solid foundation for the study of long-lasting lubrication mechanisms.

**Figure 23:**
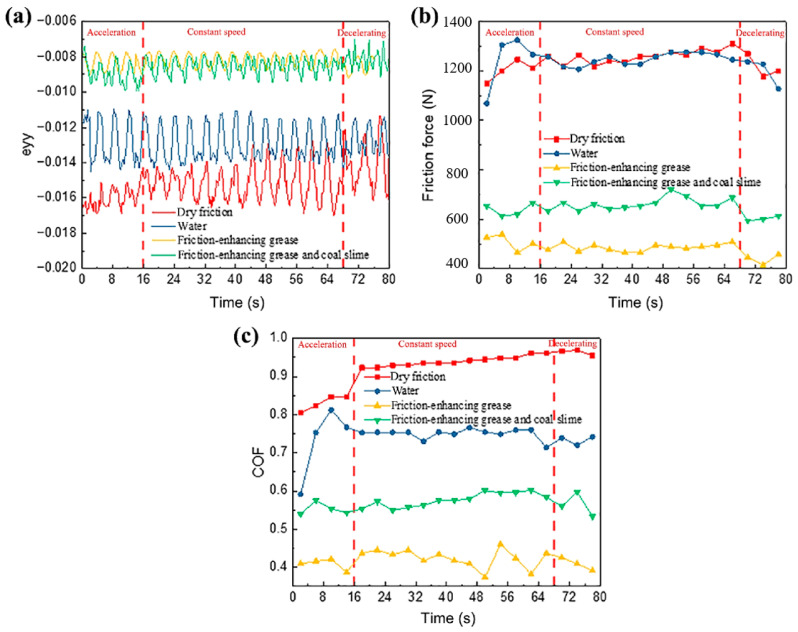
(**a**) Ystrain; (**b**) friction force; and (**c**) friction coefficient under different contact interface mediums under trapezoid acceleration, respectively [27].

A synergistic study of the thermodynamic behavior of the friction interface and media optimization reveals the critical role of lubrication regulation in enhancing system stability. Ma et al. [207] clarified that the sliding speed is the main factor affecting the frictional heat accumulation and fluctuation by analyzing the three-dimensional transient temperature field, which provides a theoretical basis for understanding the risk of thermal failure under high-speed operating conditions. On this basis, Guo et al. [27] further considered the actual mine dynamic working conditions, and their experiments using digital image correlation technology showed that the drag-enhancing gel-like grease could significantly reduce the strain and friction of the friction lining, and the results are shown in Figure 23, with the maximum reductions of 45.6% and 64.3%, respectively. This not only confirms the stabilizing effect of specific lubricating medium on the friction transfer performance but also extends the research perspective from pure temperature control to the comprehensive optimization of interface mechanical response and system dynamic stability. However, both studies focus on short-term performance verification and have not yet revealed the long-term performance evolution of the lubricating medium under continuous heat-force coupling and its influence mechanism on the service life of the system. Therefore, constructing prediction models that can simultaneously couple transient thermal behavior, media property degradation, and long-term wear evolution is a core challenge to achieve the leap from condition monitoring to active reliability management of enhancement systems.

**Figure 24:**
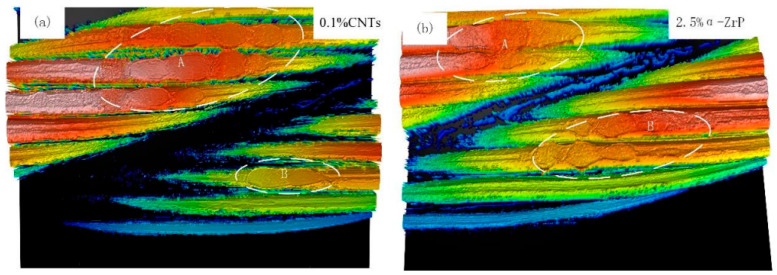
Comparison of two wire rope scars (**a**) Wear scar of steel wire rope with CNTs as additive (**b**) Wear scar of steel wire rope with α-ZrP as additive [208].

To further enhance the anti-wear properties of gel-like grease, additives are typically incorporated into the formulation to improve its performance. The use of nano-additives to enhance the performance of gel-like grease has been the focus of research on the synergistic and competitive effects of different additives, but the “optimal formulation” is highly dependent on the specific operating conditions. For example, a comparative study by Zhou et al. [208] showed that the friction-reducing and anti-wear effect of the layered silicate α-ZrP was better than that of carbon nanotubes (CNTs) under the sliding wear conditions of steel wire ropes in mines, and the optimal mass fraction was 2.5%. This finding contrasts with the findings of Sun et al. [187], who found that the compounding of 1 wt% multi-layer graphene with 1 wt% micronized graphite in the base grease was most effective in mitigating micromotor wear of steel wires. This inconsistency highlights the critical impact of wear form (sliding vs. micromovement) on additive performance, suggesting that there is no universal “optimal additive” and that future research needs to be more focused on establishing an “additive-operating condition-performance” mapping relationship, rather than seeking a “one-size-fits-all” solution. Future research should be more devoted to establishing the mapping relationship of “additive-working condition-performance” rather than seeking a single universal solution. Figure 24 shows the optimal ratio of the two additives obtained by the three-dimensional morphology of the abrasion marks, it is observed that the laminated material α-ZrP is still better friction and anti-wear effect, the area of its abrasion marks is smaller, where the abrasion marks are located in the steel wire surface of the plastic deformation is also small.

In summary, grease lubrication is an effective method for reducing sliding wear in steel wire ropes, though its mechanism of action and influencing factors warrant further investigation. By selecting appropriate gel-like greases, optimizing lubrication methods, improving wire rope structures, and developing novel gel-like greases, sliding wear can be significantly reduced under grease lubrication. This approach extends the service life of steel wire ropes and ensures the safe operation of equipment. In the future, with the development of new materials and technologies, research on sliding wear of steel wire ropes under grease lubrication will achieve greater progress, providing more reliable guarantees for the safe use of steel wire ropes. The research of the aforementioned scholars is synthesized in Table 7, detailing their specific studies on the fretting and sliding wear of wire ropes under gel-type lubricant conditions.

### 3.4. Structural Integrity and Deformation Behavior of Gel-like Greases

The positive effects of gel-like grease in improving the performance of steel wire ropes and inhibiting micro-motion and sliding wear were summarized in the previous paper. However, an in-depth understanding of their intrinsic structural integrity and dynamic deformation behavior is necessary to achieve their long-lasting lubrication and guide the development of high-performance gel-like greases [209].

The structural integrity of the gel-like grease is the physicochemical basis for its performance of the lubrication and protection functions, the core of which lies in the three-dimensional networked soap fiber skeleton constructed by the thickening agents (e.g., lithium soaps, calcium composite sulfonate, etc.). This microscopic skeleton directly determines the macroscopic mechanical stability and adhesion ability of the grease by adsorbing and fixing the base oil. As mentioned in Section 3.1, a structural integrity and stability of the gel system, to ensure that the grease in high loads, shock vibration, and other complex conditions, effective resistance to mechanical centrifugal force, and gravity, to prevent its premature loss and base oil precipitation, so as to maintain a layer of complete and continuous lubrication film on the surface of the steel wire ropes and strand gap. However, the structure has faced serious challenges in service. Under the coupling effect of continuous mechanical shear (e.g., relative slip between strands during repeated bending of steel wire ropes) and high temperatures (caused by frictional and ambient temperatures), the soap fiber skeleton undergoes irreversible fracture, untwisting, and even oxidative hardening, a process commonly referred to as “shearing”. Once the structure is damaged, the colloidal system of grease tends to collapse, and the immediate consequence is a sharp drop in adhesion, leading to lubrication film rupture and localized dry friction. This will not only instantly exacerbate the wear and tear, but also in the steel wire surface triggered by severe stress concentration, fatigue cracks and the expansion of the source, and due to the failure of the protective barrier to significantly accelerate the corrosion process of the steel wire.

Therefore, the structural integrity of a grease is not a static indicator, but a dynamic core that determines the evolution of its performance throughout its service life. It is not only a key parameter to measure the life of the grease itself, but also one of the fundamental factors that directly govern the fatigue life and corrosion resistance of steel wire ropes. Research on the structural stability of different grease formulations is the core of realizing long-lasting and reliable lubrication and predictive maintenance of steel wire ropes.

Steel wire ropes are subjected to bending, stretching, and vibration during operation, resulting in the internal gel-like grease being in a complex state of dynamic deformation. Their rheological properties (e.g., viscoelasticity, thixotropy) play a key role in this process. Under small amplitude (such as micro wear), high viscosity gel-like grease may not be able to flow sufficiently to the contact area, resulting in insufficient lubrication; and under large deformation (such as when rounding the pulley), the shear-thinning properties of the gel-like grease make its viscosity drop temporarily, which is conducive to penetrate into the interior of the steel wire ropes, but if the recovery is poor, then it will lead to local lubrication film thinning. As shown in a number of studies in Section 3.2 and Section 3.3, the tribological properties of gel-like grease under different deformation conditions are very different, the fundamental reason is that the deformation behavior changes the thickness, uniformity, and load-bearing capacity of the lubricant film, which directly affects the sliding and kinematic properties of the steel. Steel wire ropes in the operation of the bending, stretching and vibration load, so that its internal gel-like grease in a complex multi-mode dynamic deformation state. In this process, the rheological properties of the grease—especially its viscoelasticity and thixotropy—play a decisive role in the lubrication effect. Under micro-wear conditions with small amplitudes, the high viscosity gel-like grease is unable to flow adequately into the contact area due to its significant elastic response, resulting in inadequate interfacial lubrication and accelerated localized wear. On the contrary, when the steel wire ropes around the pulley experience large deformation, strong shear will trigger the shear thinning effect of the gel-like grease, so that its apparent viscosity is temporarily reduced, this feature is conducive to the gel-like grease to the internal penetration of the steel wire rope, but the subsequent structure of the ability to restore the crucial: if the poor recovery will lead to local lubrication film is too thin or even rupture, cannot form an effective protection.

As shown in the studies of Xu et al. [150] and Peng et al. [206] in Section 3.2 and Section 3.3, the tribological properties of gel-like grease under different deformation modes differ significantly, and the underlying mechanism is that the dynamic deformation directly regulates the thickness, homogeneity, and load-carrying capacity of lubricating film. Therefore, to assess the performance of gel-like grease, it is necessary to go beyond the static indicators and examine its rheological response and film-forming properties under dynamic deformation that simulates real working conditions, which is of core significance for predicting the life and reliability of steel wire ropes in complex service environments.

### 3.5. Performance Limitations and Application Challenges of Lubrication Technology

Performance optimization of gel-like grease is essentially a complex multi-objective trade-off process, and it faces multiple technical bottlenecks in engineering applications. These limitations not only affect the lubrication effect, more directly related to the overall service safety of steel wire ropes.

The design of gel-like grease formulations is faced with a number of mutually constraining technical requirements. In terms of rheological properties, in order to improve the high temperature adhesion and increase the consistency, often at the expense of low-temperature fluidity and permeability, which leads to the cold conditions of gel-like grease is difficult to effectively penetrate to the internal contact area of steel wire ropes. In terms of chemical performance, there is a significant contradiction between the pursuit of extreme pressure anti-wear performance and environmental requirements: although the extreme pressure additives containing sulfur, phosphorus, and other active elements can effectively enhance the load-carrying capacity, their environmental compatibility is poor, and it is difficult to meet the increasingly stringent environmental regulations.

The performance decay of gel-like grease in the process of long-term service is a key factor affecting the life of steel wire ropes. Under the synergistic effect of mechanical shear, thermo-oxidative aging and environmental contaminants (moisture, mineral dust, etc.), the soap fiber structure of the gel-like grease is irreversibly damaged, resulting in base oil precipitation and additive depletion. This structural degradation leads to a systemic failure of the lubrication function—base oil loss leads to insufficient lubrication, changes in consistency affect adhesion properties, and additive depletion reduces extreme pressure protection. There is a lack of reliable models that can accurately predict the evolution of a grease’s performance over its entire life cycle.

Specific application scenarios place nearly contradictory demands on the performance of gel-like greases. In mining friction hoisting systems, grease needs to provide excellent friction reduction properties to protect the steel wire ropes but must also maintain a sufficient coefficient of friction to ensure effective power transfer between the ropes—sheaves. In the field of offshore engineering, gel-like greases have to resist salt spray corrosion as well as maintain the integrity of the lubricant film under dynamic loads. These opposing technical requirements greatly increase the difficulty of formulation design, often requiring complex additive package systems to achieve a balance of performance.

It is worth noting that there is a close correlation between the performance limitations of gel-like greases and the inadequacy of testing techniques. Damage to steel wire ropes caused by the failure of gel-like grease often occurs in internal areas where detection is difficult, and detection may be affected by the presence of grease, which affects detection accuracy. This negative correlation effect between technologies highlights the systemic flaws in the current technology system. The future research and development of gel-like grease should break through the traditional idea of single performance optimization, dedicated to the development of intelligent lubrication materials with self-repairing, environmental response characteristics, and the establishment of accurate life prediction model based on the actual working conditions, in order to promote the lubrication technology from the passive protection to the active protection of the leap.

## 4. Summary and Outlook

### 4.1. Summary

This paper provides a systematic review of the failure mechanisms of steel wire ropes and the research progress on the anti-friction effects of gel-like greases on steel wire ropes. The discussion primarily focuses on the failure modes of steel wire ropes, their detection methods, and the impact of these factors on the performance of steel wire ropes.

(1) Current research has fully revealed the independent mechanism of wear, fatigue and corrosion as the main failure modes of steel wire ropes. However, this review emphasizes that the focus of future research must shift to understanding the interaction and competition of these modes under multi-field coupled loading (e.g., tensile-flexure-torsion-vibration). The establishment of a physically based “micro damage-macro performance” correlation model is a fundamental prerequisite for realizing the transition from passive after-action analysis to active life prediction, which will provide a theoretical cornerstone for the development of the next-generation high-precision digital twin system.
(2) Although all kinds of detection technology (such as magnetic leakage, acoustic emission, machine vision) have been greatly developed, any single technology has its own inherent perceptual blind spot and application limitations. Therefore, the future technological breakthrough lies not only in the sensor itself, but also in the construction of an intelligent detection system based on the failure mechanism and lubrication status as the basis for decision-making. Specifically, the development of the detection strategy should be a two-stage decision-making process: firstly, the dominant failure modes (e.g., wear, fatigue, or corrosion) are judged according to the working conditions and historical data, so as to select the most sensitive core technology (e.g., leakage detection is used to quantitatively assess wear and tear, and acoustic emission is used to monitor the fatigue activity); secondly, the immediate lubrication state of the steel wire ropes (grease film uniformity, drying out, or serious contamination) must be used for the feasibility and accuracy of the technique to be corrected and compensated in real time. On this basis, by combining heterogeneous information reflecting different failure mechanisms, such as mechanics, magnetism, acoustics, etc., with deep learning algorithms, we can ultimately realize a leap in capability from “defect identification” to “health state assessment” and “remaining lifetime prediction” to form accurate risk-based maintenance decisions. It is only through the combination of deep learning algorithms that we can ultimately realize the leap from “defect identification” to “health condition assessment” and “remaining life prediction” and form accurate risk-based maintenance decisions.
(3) Gel-like grease is not only a wear-reducing medium, but also a key functional material that determines the long-term reliability of the steel wire ropes system. Beyond the traditional performance evaluation, the future of lubrication technology will be “intelligent” and “functional” direction. This includes the development of smart lubrication materials with self-healing, environment-responsive (e.g., pressure, temperature) properties, as well as exploring the long-lasting mechanism of nano-additives under extreme operating conditions. The ultimate goal is to transform the lubrication system from a passive consumable to an active protection unit that senses the interface state in real time and dynamically adjusts its tribological behavior.

### 4.2. Outlook

This paper reviews the analysis of wire rope failures and the research on the anti-friction effects of gel-like grease on wire ropes. Although significant progress has been made in these areas, several unresolved issues and future research directions remain.

(1) Although the article separately discusses three failure modes—wear, fatigue, and corrosion—it remains relatively weak in analyzing multi-factor coupling effects and lacks systematic models to reveal their interactive mechanisms. Further exploration is needed into failure mechanisms under multi-factor coupling, such as the synergistic effects of wear-fatigue-corrosion, to establish more precise life prediction models. Developing more efficient nondestructive testing techniques, such as real-time monitoring systems based on artificial intelligence, is essential for achieving dynamic assessment of wire rope health status. Develop new high-performance lubricants, such as nano-composite lubricants and self-repairing lubricating films, to meet the challenges of extreme operating conditions (such as ultra-low temperatures, high humidity, and highly corrosive environments). Study the long-term performance evolution of lubricating grease, optimize lubrication cycles and application techniques and ensure the durability and uniformity of lubrication effects.
(2) Long-term performance evolution of gel-like grease and intelligent lubrication strategy. The current research on the evolution of the performance of gel-like grease in the long-term service process is still insufficient understanding, especially in the multi-factor coupling under the failure mechanism needs to be revealed. In order to achieve long-term reliable lubrication, future research is facing three major challenges: first, the balance between mechanical stability and lubrication performance, i.e., enhancement of structural integrity, may be sacrificed at the expense of its low-temperature mobility and permeability, resulting in uneven lubrication; second, the mechanism of multi-factorial coupled aging is not clear, the grease is subjected to mechanical shear, oxidation, thermal aging, and the intrusion of environmental contaminants in the actual conditions, and the synergistic effect of these factors accelerates the performance decay. The synergistic effect of these factors accelerates the deterioration of performance, but the related research is not yet in-depth; third, the lack of intelligent monitoring and adaptive lubrication strategies. Therefore, in the future, efforts should be made to develop intelligent gel lubrication materials with self-repairing function and establish accurate lubrication life prediction model based on actual working conditions, so as to promote the lubrication technology from passive maintenance to the intelligent prediction and adaptive protection of the leap.
(3) Based on the systematic knowledge established in this review, the future research paradigm of steel wire ropes safety technology will see a fundamental shift from reactive maintenance to intelligent predictive maintenance. This shift is mainly centered on three cutting-edge directions: first, the development of intelligent lubrication materials, the key lies in upgrading grease from a static protection medium to an “intelligent functional unit” that can sense pressure and temperature and dynamically adjust its tribological properties, for example, through the integration of nano-additives (e.g., functionalized graphene, molybdenum disulfide) to achieve on-demand repair and wear reduction. Secondly, it is a hybrid monitoring system with multi-physical field fusion, which requires breaking through the limitations of a single detection technology and building a sensing network that can capture all aspects from microscopic damages to macroscopic defects through the synergistic integration of leakage magnetism, otoacoustic emission, ultrasound, and machine vision sensors; and ultimately, the convergence of the above directions lies in the AI-driven digital twin and decision-making system, which is to utilize advanced algorithms, such as deep learning, to analyze the performance of the hybrid system, and to make decisions. Deep learning and other advanced algorithms are used to fuse and analyze the massive data acquired by the hybrid monitoring system and build a high-fidelity digital twin model of steel wire ropes on this basis, so as to realize the dynamic prediction of the remaining service life and accurate maintenance decision-making based on risk, and ultimately to form a closed-loop intelligent management system that is self-aware, self-decision-making, and self-optimizing.

## Figures and Tables

**Table 1:** Comparison table of the position of two broken wire points of the wire rope.

Experimental Group Number	First Breakage/cm	Second Breakage/cm	Distance Between Two Points/cm	Two Error Values/cm	Error/%
1	23.37	43.57	20.17	−0.03	0.15
2	25.02	45.17	20.15	−0.05	0.25
3	30.99	51.12	20.13	−0.07	0.35
4	39.95	60.26	20.31	0.11	0.54
5	42.91	63.23	20.32	0.12	0.59
6	62.03	82.32	20.29	0.09	0.45
7	55.31	75.42	20.11	−0.09	0.45
8	70.58	90.69	20.11	−0.09	0.45
9	66.58	86.75	20.17	−0.03	0.15
10	56.58	76.73	20.15	−0.05	0.25
	mean error			±0.073	0.36

**Table 2:** Describes researchers’ specific studies on steel wire rope failure detection methods.

Reference Number	Author(s), Year	Major Findings
[116]	E, Mnaka. et al., 2021	It was found that bending had the greatest impact on the performance of steel wire ropes under conditions of stretching, bending, and twisting.
[118]	G, Olszyna. et al., 2022	A new steel wire rope inspection method (magnetic particle inspection) has been proposed.
[21]	P, Zhou. et al., 2023	A method combining finite element analysis and mechanical tension analysis was proposed to analyze surface wire breakage failure.
[119]	J, Zhang. et al., 2023	Quantitative detection of structural damage in steel wire ropes using a new magnetic coupling theory model.
[120]	J, Zhang. et al., 2023	The correlation between wire rope damage signals and cross-sectional loss rate (CLR) was discovered.
[121]	Q, Chen. et al., 2023	Proved the effectiveness of weak magnetic field excitation on the magnetic memory signal of steel wire ropes.
[126]	Z, Hu. et al., 2023	A fatigue damage detection method (using a nondestructive testing instrument to collect signals) has been verified.
[122]	M, Ren. et al., 2023	A magnetic leakage detection device and magnetic focusing device were designed to reduce steel wire rope detection costs and reduce steel wire rope detection components.
[123]	J, Han. et al., 2024	Residual networks have been found to be effective in identifying internal and external wire breakage faults in steel wire ropes.
[124]	X, Zhao. et al., 2024	A MFL model for detecting internal defects in steel wire ropes was proposed and verified with a test error of less than 10%.
[125]	Z, Zhou. et al., 2023	A fracture detection device based on multiple principles was designed to solve the problem of nondestructive testing of steel cables in high-speed elevators.
[127]	T, Krakowski. et al., 2021	A magnetic method has been discovered that can be used to detect wear on mixed-load steel wire ropes (steel wire rope + belt).
[128]	Q, Chen. et al., 2023	A steel wire rope broken wire damage detection system capable of damage identification, location, cutting, and classification has been designed.
[129]	N, Yao. et al., 2025	An online intelligent nondestructive testing instrument with an average error of 0.36% has been developed.
[130]	D, Lu. et al., 2024	A multi-channel, high-precision MBF filter transformer probe has been designed.
[131]	S, Liu. et al., 2022	A new MFL imaging and quantitative defect identification method is proposed.
[132]	T, Huang. et al., 2024	A nondestructive testing instrument combining wireless remote control and real-time online functions has been developed.
[133]	Y, Bao. et al., 2024	A new algorithm for nondestructive testing of steel wires at high altitudes (VovNetV3.5) has been proposed, and it has been proven that the highest accuracy can reach 0.975.
[134]	H, Wang. et al., 2024	A differential signal detection device utilizing a double-layer sensor was designed, and the accuracy of Xi’an defect detection was improved by 3.15%.
[135]	Z, Yu. et al., 2024	A real-time wire rope broken wire detection system has been designed for mining applications, with performance indicators that surpass those of other mainstream detection algorithms.
[136]	W, Liu. et al., 2024	A test platform simulating the operating conditions of steel wire ropes was designed to solve the problem of damage to detection probes caused by high-speed operation of magnetic yoke detection equipment in complex environments.
[137]	P, Zhou. et al., 2023	A visual inspection system for the healthy operation and maintenance of heavy water reactors has been developed, with an average detection accuracy of 82.3%.
[138]	W, Zhenbin. et al., 2024	A method for detecting surface defects on WR surfaces based on the deep learning models YOLOv8s and U-Net was proposed, with detection results improved by 1.13% compared to the basic model.

**Table 3:** Summarizes a comparison of several steel wire rope testing methods.

Method	Measurement Principle	Advantages	Disadvantages
Appearance inspection	Manual visual slow inspection of surface defects	Simple and direct surface damage assessment	Time-consuming, and results are affected by human factors
Magnetic particle inspection	Measurement of leakage flux	Qualitatively determine wire breaks, rust, wear, and other defects	Difficult to quantitatively measure and distinguish coexisting defects
Ultrasonic testing	Ultrasound propagation in a medium	Detect short lines, single point detection distance is long	Weak noise resistance, unable to accurately reflect defect conditions
Eddy current testing	Eddy current effect	Detect wire breakage and rust	low signal-to-noise ratio
Acoustic emission testing	Measuring ultrasonic waves generated by changes in steel wire rope structure	Detection of steel wire rope breakage and deformation	Can only be used for static load components, low signal-to-noise ratio, high instrument cost, difficult to measure dynamically
Mechanical performance testing	Determine the strength indicators of steel wire ropes through mechanical tests such as stretching, bending, and twisting.	Directly reflects the actual load-bearing capacity of steel wire ropes, with intuitive and reliable results	This is a destructive test, only applicable to sampling tests, with a long test cycle and high cost
Machine vision inspection	Images are captured using a high-definition camera and combined with image processing algorithms to automatically identify defects, such as broken threads, and quantify defect parameters.	Highly automated, capable of continuous detection, and highly efficient	Skills detect surface defects but cannot identify internal damage; greatly affected by lighting conditions, oil stains, and dust, prone to misjudgment

**Table 4:** Guidelines for the application of the main test methods for steel wire ropes under different grease lubrication conditions.

Detection Methods	Sensitivity to Lubrication Status	Limitations	Approximate Cost	Applicable Scenarios
Exterior Inspection	High. Uniform grease film interferes with observation; dried/flaked gel-like grease can completely mask surface defects such as broken filaments, corrosion, etc., leading to serious misjudgments or missed inspections.	Highly subjective, unable to detect internal defects, inefficient, dependent on personnel experience.	Low	Initial screening. Suitable for rapid inspection of surfaces with visible wire breakage, deformation, and severe corrosion in any state of lubrication.
Magnetic leakage detection	Medium. Homogeneous lipid films have almost no effect; thick, uneven layers of dried lipids attenuate the magnetic field and produce pseudo-signals [120,131]; they tend to magnetize unevenly at high speeds [129].	Insensitive to defects parallel to the wire, difficult to distinguish between types of defects, affected by lift-off effects.	Moderate	Routine in-service inspection. For well-lubricated or cleaned steel wire ropes, dedicated to the quantitative detection of wire breakage and loss of section (LMA) [126,132].
Ultrasonic testing	High. Layers of grease (especially dried grease) can severely impede incoming sound waves, resulting in severe signal attenuation or complete failure.	Requires good coupling agent, sensitive to surface roughness, weak noise immunity, slow detection speed.	Middle to high	Critical part sampling. Usually used after cleaning grease to detect internal corrosion, cracks, and core condition.
Machine vision	Extremely high. Oil and grease coverings are the main sources of interference, which can completely mask the target features and cause the algorithm to fail [140].	Surface defects only, highly affected by light and occlusion, complex algorithm development	High initial investment	Surface quality monitoring. For clean or well-lubricated steel wire ropes for automatic identification of surface wire breakage and wear, e.g., healthy O&M of heavy water pile wire ropes [140].
Acoustic emission	Low. The grease layer has a low impact on stress wave propagation, making it suitable for real-time online monitoring under lubrication.	Low positioning accuracy, susceptible to environmental noise, difficult to recognize defect types, expensive instruments	honorific	Real-time safety monitoring. Early warning of active defects (fatigue cracks, broken wires) in critical areas (e.g., ropeways, bridges) under various lubrication conditions.

**Table 6:** Lubrication characteristics of steel wire ropes in different application environments [82,149,151,152,153,157,159].

Application Scenarios	Environmental Characteristics	Lubrication Requirements	Recommended Gel-Like Lubricant
Mine hoists Mining equipment Conveyor systems, etc.	High dust High humidity Heavy loads and impacts	High adhesion Extreme pressure anti-wear properties Anti-rust properties	High viscosity extreme pressure lithium-based grease Mining steel wire rope lubricating grease containing graphite/molybdenum disulphide
Port cranes Ship mooring ropes Offshore lifting platforms, etc.	Salt spray corrosion High humidity Dynamic load	Salt spray corrosion resistance Water erosion resistance Good low temperature adaptability	Calcium sulfonate-based grease Grease specially designed for steel wire ropes resistant to seawater corrosion
Tower crane Bridge crane Construction hoist, etc.	Outdoor exposure Frequent bending High load	Antioxidant properties Permeability Friction enhancement properties	Polyurea-based grease Highly penetrating lithium-based grease
Elevator traction steel wire rope Cable car ropeway, etc.	Low noise requirements Frequent start–stop operation Environmental performance	Low friction coefficient Long service life No pollution	Polyalphaolefin synthetic grease Environmentally friendly polyether grease
Extreme environments	High temperature Extreme cold Chemical corrosion environment	High temperature resistance (>150 °C) Low temperature resistance (<−30 °C) Acid and alkali resistance	Composite aluminum-based high-temperature grease Low-temperature synthetic grease Polytetrafluoroethylene grease

**Table 7:** Describes the researchers’ specific studies on micro-motion and sliding wear of steel wire ropes under lipid lubrication.

Reference Number	Author(s), Year	Major Findings
[174]	C, Xu. et al., 2019	The effects of two factors, wire rope contact pressure, and micro-motion amplitude, on the micro-motion wear mechanism of wire ropes were discussed.
[175]	K, Huang. et al., 2025	The effects of three factors—wire rope contact load, micro-motion amplitude, and cycle count—on the micro-motion wear mechanism of wire ropes were discussed.
[176]	Y, Chen. et al., 2023	A method for predicting wire rope wear marks by simulating wear marks has been proposed.
[177]	D, Wang. et al., 2021	Through research on the micro-motion fatigue of steel wire ropes under different parameters, the relationship between mine hoisting parameters, micro-motion wear degree, and micro-motion fatigue wear life was discovered.
[178]	M, Imran. et al., 2025	The micro-motion wear mechanism of steel wire ropes under acidic conditions was studied.
[180]	Z, Hu. et al., 2023	Using gel-like lubricants, such as grease and graphite, can reduce micro-motion friction wear on steel wire ropes.
[184]	C, Xu. et al., 2021	It was discovered that adding ore particles under steel wire rope grease lubrication conditions accelerates steel wire failure.
[186]	C, Dyson. et al., 2020	A cross-cylinder reciprocating micro-motion wear test method has been developed to determine key lubrication parameters and evaluate lubrication technology.
[187]	Z, Sun. et al., 2020	The four-ball friction test demonstrated that multi-layer graphene-activated micron-sized graphite enhances the anti-wear capability of the base gel-like grease.
[188]	G, Wang. et al., 2021	The coefficient of friction and friction temperature rise exhibit a positive correlation when lubricating steel wire ropes with multi-layer graphite gel-like grease.
[190]	K, Huang. et al., 2021	Adding mineral particles to a simulated mine grease-lubricated steel wire rope environment increases micro-motion wear on the steel wire rope and causes more cutting grooves and breakage pits to appear.
[205]	S, Xue. et al., 2019	A model capable of predicting sliding wear and fatigue damage in steel wire ropes has been discovered.
[206]	Y, Peng. et al., 2018	It was discovered that under gel-like grease lubrication, the temperature rise of steel wire ropes is more significantly affected by sliding friction than in lubricated conditions.
[207]	W, Ma. et al., 2018	It has been verified that sliding speed has a significant impact on the average temperature rise of steel wire ropes.
[27]	Y, Guo. et al., 2025	It was found that using drag-enhancing gel-like grease can significantly reduce the maximum strain and friction force of steel wire ropes.
[208]	X, Zhou. et al., 2021	It was discovered that adding the additive α-ZrP to gel-like lubricating grease for mining steel wire ropes yields better anti-wear and friction-reducing effects than CNTs.

## Data Availability

The original contributions presented in this study are included in the article. Further inquiries can be directed to the corresponding author.
